# Dynamic spatiotemporal graph attention networks for cross-regional multi-disease forecasting and intervention optimization

**DOI:** 10.3389/fpubh.2026.1720620

**Published:** 2026-02-04

**Authors:** Siyan Liu, Lixing Cao

**Affiliations:** 1 Beijing University of Chinese Medicine, Beijing, China; 2 Huai'an Second People's Hospital, Huai'an, Jiangsu, China

**Keywords:** cross-regional transmission, equity-focused public health decision-making, intervention optimization, multi-disease forecasting, spatiotemporal graph attention network

## Abstract

**Introduction:**

Accurately predicting cross-regional spread of infectious diseases and designing cost-effective interventions is challenging due to population mobility, multi-pathogen circulation, and spatiotemporal heterogeneity. This study aims to build a unified framework that improves multi-disease forecasting, enhances interpretability of transmission pathways, and enables data-driven optimization of public-health interventions.

**Methods:**

We develop a spatiotemporal graph attention network (ST-GAT) that integrates surveillance, meteorological, healthcare, and NPI data on a dynamic multi-relational graph combining geographic adjacency and origin-destination mobility. Spatial and temporal attention with a distribution-aware NB/ZINB decoder generates calibrated 1–4-week probabilistic forecasts, and the model is embedded in a multi-objective optimization engine to evaluate vaccine allocation and mobility restriction strategies under cost, fairness, and feasibility constraints.

**Results:**

Using ILI, HFMD, dengue, and RSV data, ST-GAT reduces MAE (34% vs ARIMAX, 27% vs Prophet, 15% vs LSTM/GRU) and improves WIS/CRPS across diseases. Spatial attention identifies high-weight transmission corridors, temporal attention highlights short lags of 1–4 weeks, and optimization shows a vaccine-first strategy achieves the best cost-effectiveness and stability.

**Discussion:**

The framework provides an integrated, interpretable, and generalizable solution for real-time epidemic prediction and equitable public-health decision-making.

## 1 Introduction

The rapid increase in global interconnectedness, urbanization, and interregional mobility has profoundly reshaped the transmission dynamics of infectious diseases. Respiratory, enteric, and vector-borne pathogens now spread across administrative boundaries more quickly and more frequently, creating overlapping epidemic waves that pose substantial challenges to surveillance and response systems (1, 2). Traditional prediction approaches—whether time-series models or mechanistic epidemic models—are typically designed for a single disease and a single region, making them insufficient for capturing multi-pathogen co-circulation, spatial spillovers, and dynamic changes in contact structures. Moreover, interventions such as vaccination, school closures, and mobility restrictions often exhibit uneven effectiveness across regions, further complicating policy design (3–5). These emerging challenges underscore the need for modeling frameworks capable of integrating multi-source data, capturing spatiotemporal dependencies, and linking predictions directly to optimized intervention strategies. Motivated by these gaps, our study proposes a unified spatiotemporal graph-based approach to characterize cross-regional transmission processes and support data-driven public-health decision-making. Recent public-health research further emphasizes that timely and interpretable predictive models are essential not only for outbreak forecasting, but also for supporting evidence-based decision-making, intervention prioritization, and equitable resource allocation at population scale (6, 7).

The cross-regional spread of infectious diseases has become a major challenge to global public health. With the intensification of globalization, urbanization and population mobility, respiratory diseases (such as influenza, RSV), intestinal diseases (such as hand, foot and mouth disease) and mosquito-borne diseases (such as dengue fever) have spread rapidly between different regions, causing serious health burdens and socioeconomic losses (2, 3). Especially after the outbreak of the new coronavirus epidemic, how to achieve accurate prediction and effective intervention in a complex environment with multiple diseases and multiple regions has become an important direction of prevention and control research (4). Traditional infectious disease prediction methods are mainly based on time series (such as ARIMA, Prophet) or mechanism models (such as SEIR), which have certain applicability in single disease and single region scenarios (8, 9). However, they often have difficulty in depicting the complex network relationship between cross-regional flow and geographical proximity, and lack the ability to handle multiple diseases at the same time. In recent years, deep learning, especially time series neural networks (such as LSTM, GRU) and graph neural networks (GNNs), have gradually been applied to disease prediction (10–12). These methods can automatically extract patterns from large-scale heterogeneous data, but they still have the following limitations (13–16): (i) insufficient fusion of multi-relational networks (such as geographic adjacency + population mobility); (ii) limited probability prediction and uncertainty expression; (iii) lack of a systematic framework for policy optimization and fairness considerations (13). Recent methodological reviews highlight that, despite rapid progress in spatiotemporal deep learning, most existing studies still focus on single-disease or single-region settings and rarely integrate forecasting with downstream policy optimization or equity-aware evaluation (14–16).

**Figure 1:**
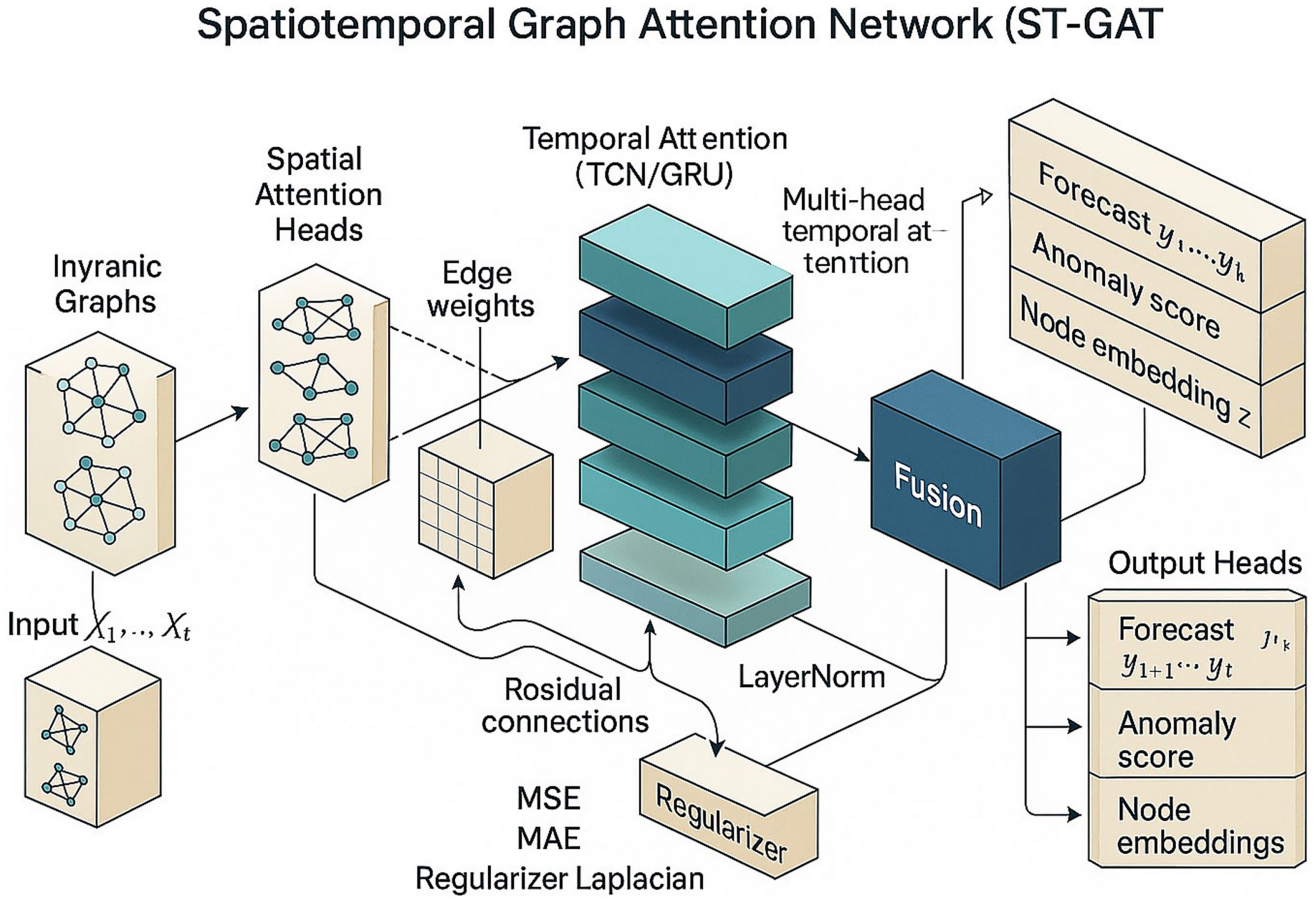
Spatiotemporal graph attention network framework.

To address the above problems, this study proposes a framework based on the Spatiotemporal Graph Attention Network (ST-GAT) to achieve dynamic transmission prediction and intervention optimization for multiple diseases and across regions (Figure 1). Its core ideas include: (1) integrating geographic adjacency and population mobility through multi-relational dynamic graph modeling; (2) combining spatial and temporal attention mechanisms to capture the main transmission channels and key lag effects; (3) using a distribution-aware decoder to output point predictions and interval predictions to provide uncertainty quantification; and (4) on this basis, establishing a multi-objective policy optimization engine to find the optimal trade-off between cost, effect and fairness.

The contributions of this study include: (i) constructing an interpretable, multi-disease ST-GAT prediction framework, significantly improving multi-horizon prediction and early warning capabilities; (ii) systematically assessing the importance of spatial, temporal, and exogenous factors, revealing cross-regional transmission mechanisms; (iii) proposing policy simulation and optimization based on the prediction model, quantifying the cost-effectiveness and fairness of strategies such as vaccines and mobility restrictions; and (iv) validating the model’s generalizability across regions and disease scenarios. These findings can provide refined prediction and optimization support for public health decision-making.

To address the limitations of existing prediction frameworks, this study proposes a unified spatiotemporal graph attention network (ST-GAT) that explicitly models cross-regional transmission pathways, short-term temporal dependencies, and heterogeneous exogenous drivers across multiple infectious diseases. The novelty of this research lies in four aspects: (i) integrating multi-relational dynamic graphs based on geographic adjacency and population mobility to quantify spatial spillover structures; (ii) introducing distribution-aware NB/ZINB decoding for calibrated multi-horizon probabilistic forecasting; (iii) providing interpretable attention mechanisms for identifying dominant transmission corridors and epidemiologically meaningful lag periods; and (iv) embedding the forecasting model into a multi-objective intervention optimization engine that jointly accounts for cost, fairness, feasibility, and epidemiological effectiveness. The dataset combines global and national surveillance systems, meteorological indicators, healthcare resource data, and policy interventions, enabling simultaneous modeling of ILI, HFMD, Dengue, and RSV transmission dynamics.

Guided by prior work on deep learning for epidemic analysis and spatiotemporal inference (e.g., bibliometric mapping of infectious disease modeling, vaccine-preventable disease dynamics, and machine-learning–assisted outbreak surveillance) (17–19), this study formulates the following research questions:

RQ1: Can a multi-disease ST-GAT framework significantly improve 1–4 week forecasting accuracy compared with classical models?

RQ2: Can spatial and temporal attention mechanisms recover epidemiologically interpretable patterns such as dominant transmission pathways and lag structures?

RQ3: How can prediction outputs be integrated with optimization methods to determine cost-effective and equitable public-health strategies?

## 2 Literature review

In recent years, research on the prediction and prevention of cross-regional spread of infectious diseases has gradually shifted from traditional statistical modeling to complex system methods based on machine learning and deep learning. Existing literature can be roughly divided into three categories: (i) traditional time series and mechanism models, (ii) time series prediction based on deep learning, and (iii) graph neural networks and spatiotemporal modeling methods.

In terms of traditional methods, earlier forecasting studies commonly rely on classical statistical time-series models such as ARIMA and SARIMA; however, recent evidence shows that deep-learning–based temporal models significantly outperform traditional approaches for nonlinear epidemic trajectories (18, 19). Recent developments integrate SEIR-type mechanistic models with graph neural networks to capture both disease dynamics and spatial interactions, forming hybrid SEIR–GNN frameworks capable of modeling multi-region transmission (20–22). However, these methods have shortcomings in characterizing spatial heterogeneity and the concurrency of multiple diseases.

In terms of deep learning methods, in recent years, recurrent neural networks such as LSTM and GRU have been used to predict influenza, dengue fever, hand, foot and mouth disease, etc. (23, 24). These methods can capture nonlinearity and long-term dependencies, but lack the ability to model spatial interactions. Some work combines CNN or Transformer models to improve temporal modeling capabilities (25). In terms of graph neural networks and spatiotemporal prediction, with the development of GNN, researchers have proposed spatiotemporal models such as ST-GCN, DCRNN, and Graph WaveNet for traffic flow prediction and gradually applied them to disease prediction (26–28). Some work uses cross-regional flow, geographic adjacency, etc. to construct dynamic graphs and combines the attention mechanism (Graph Attention Network, GAT) to improve the interpretability of transmission paths (29, 30). On this basis, some studies have attempted to couple GNN with epidemiological models such as SEIR to form a hybrid modeling method (31). In addition, fairness and policy optimization have gradually received attention. Some studies have explored the use of models to simulate vaccine distribution or flow restriction strategies, but most of them are limited to single diseases or single regions (32). Overall, existing methods still have obvious shortcomings in terms of multiple diseases, cross-regions, interpretability and policy optimization. This study further advances on the basis of the literature and proposes the ST-GAT framework to simultaneously achieve dynamic prediction and policy optimization of multiple diseases. A comparison of different method categories is shown in Table 1.

**Table 1:** Comparison and shortcomings of different method categories.

Methodology category	Typical representative	Advantage	Insufficient
Traditional timing and mechanism models	ARIMA, SARIMA, SEIR	Simple structure and good interpretability	Unable to depict spatial relationships, multi-disease scenarios are limited
Deep learning time series prediction	LSTM, GRU, CNN, Transformer	Capture nonlinearity and long-term dependence, improve prediction accuracy	Lack of modeling for cross-regional mobility and heterogeneity
Graph neural network and space–time model	ST-GCN, DCRNN, Graph WaveNet, GAT	It integrates spatial and temporal features, supports dynamic graphics, and is more explanatory	Limited research on multiple diseases and insufficient strategy optimization

Recent studies published in 2024 further reflect the rapid expansion of data-driven methodologies and evaluation practices across health-related modeling. For example, a recent Axioms study proposed a time-series ensemble technique for outbreak forecasting and highlighted the continued value of ensemble-based baselines when epidemic trajectories are nonlinear and noisy (14). In addition, a 2024 meta-analysis in World Journal of Microbiology and Biotechnology synthesized evidence across large-scale studies and demonstrated how systematic aggregation can quantify intervention effectiveness and identify heterogeneity drivers, reinforcing the importance of rigorous evidence synthesis alongside predictive modeling (15). In parallel, Heliyon reported a robust feature-selection framework for high-dimensional health datasets, emphasizing that careful variable selection and robustness to outliers can materially affect generalization and reliability in applied health analytics (16). Collectively, these recent works motivate updating the literature base and further support our emphasis on robust modeling, comparative evaluation, and generalizable decision support.

## 3 Method

### 3.1 Study design

In this study, multi disease dynamic prediction refers to the simultaneous forecasting of multiple infectious diseases-namely influenza-like illness (ILI), hand, foot and mouth disease (HFMD), dengue, and respiratory syncytial virus (RSV)-across shared spatiotemporal structures. This framework focuses on joint prediction across diseases and regions, rather than modeling within host coinfection processes.

Although these diseases differ in transmission mechanisms and epidemiological characteristics, they share common exogenous drivers such as meteorological conditions, population mobility, healthcare capacity, and non-pharmaceutical interventions (NPIs). To leverage these shared drivers while preserving disease-specific incidence dynamics, we adopt a multi-task modeling strategy in which spatial and temporal representations are learned jointly, and disease-specific incidence processes are decoded separately using appropriate probabilistic likelihoods.

**Figure 2:**
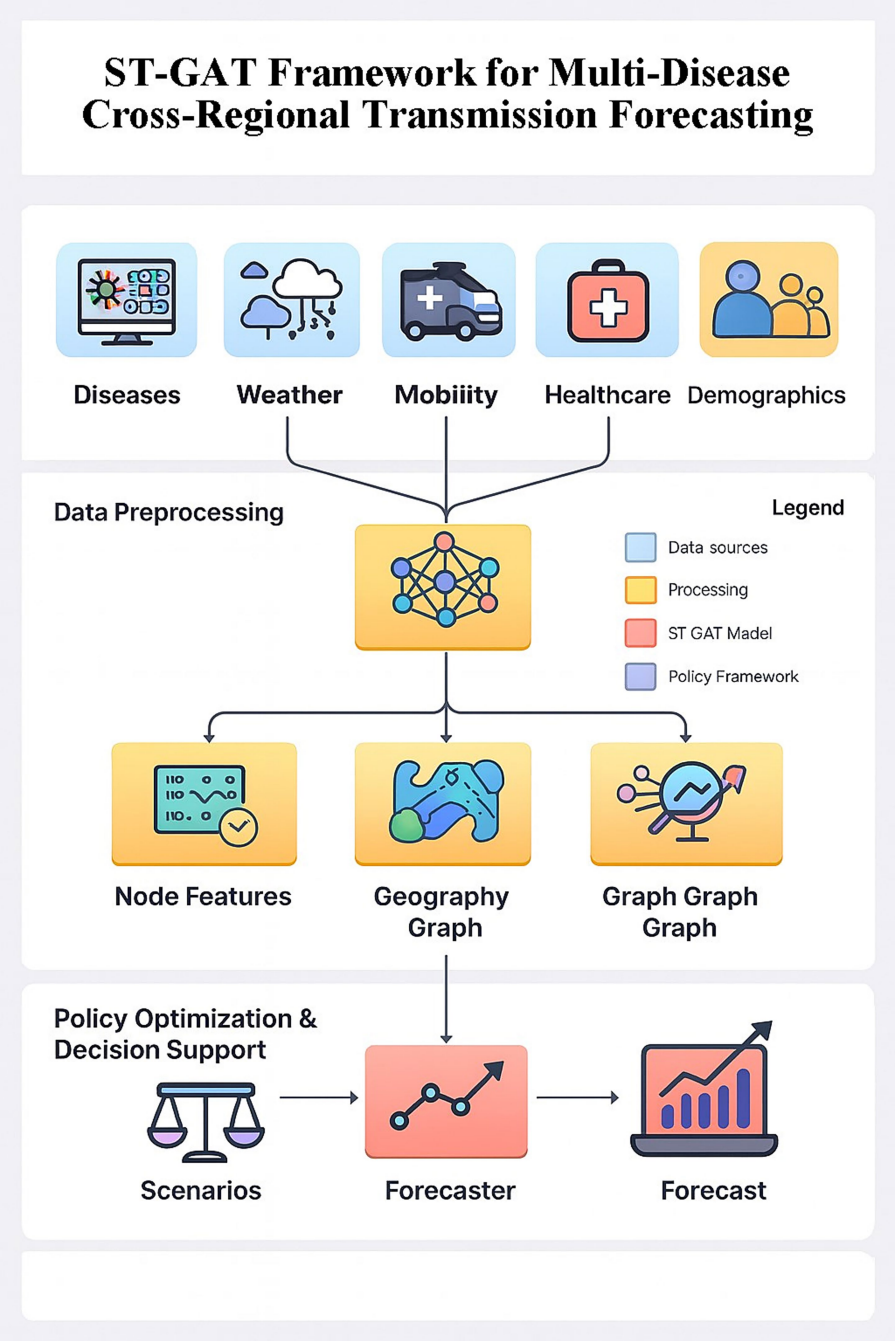
Conceptual linkage between epidemiological processes and the ST-GAT architecture. **(A)** Epidemiological dynamics layer. **(B)** Multi-relational dynamic graph layer. **(C)** ST-GAT computational layer with spatial and temporal attention. **(D)** Distribution-aware forecasting and policy layer.

We construct a spatiotemporal graph attention network (ST-GAT) to support multi-scale, multi horizon forecasting and downstream policy optimization. The overall workflow is illustrated in Figure 2.

Let y_{i,t}(d) denote the newly confirmed number of cases of disease d in region *i* during epidemiological week *t*, and let x_{i,t}represent the corresponding node-level feature vector. Given information ℱ-t available up to week *t*, the objective is to generate distributional and quantile forecasts for future horizons h. The formal mathematical definition of the forecasting task is given in Equation 1, with full notation and derivation provided in Supplementary Methods S1.

### 3.2 Data sources and preprocessing

The data for this study were obtained from open databases and licensed health surveillance systems. The open source data are fully reproducible, while the contracted data were used to enhance strategy optimization and equity analysis.

1. For case surveillance, influenza (ILI) data were obtained from the WHO FluNet/FluID weekly surveillance database,^1^ respiratory syncytial virus (RSV) data were obtained from the US CDC NREVSS and RSV-NET,^2^ dengue data were obtained from the OpenDengue global open database,^3^ and hand, foot and mouth disease (HFMD) data were obtained from the National Institute of Infectious Diseases (NIID) weekly reports of Japan^4^ and the Taiwan CDC open data.^5^
2. Population mobility data was obtained using ODT Flow Explorer^6^ and combined with Movement Range data from Meta Data for Good.^7^
3. The geographic adjacency matrix was constructed based on the GADM administrative division vectors.^8^
4. Meteorological data was obtained from the NOAA National Centers for Environmental Information (NCEI) Climate Data Online,^9^ and air quality data was obtained from the OpenAQ global open platform.^10^
5. Regarding policies and interventions, the intensity of policies during COVID-19 was measured using the Oxford COVID-19 Government Response Tracker (OxCGRT).^11^
6. Population and medical resource characteristics are from the United Nations World Population Prospects^12^ and the World Bank World Development Indicators,^13^ respectively.

Data sources and fields are standardized according to Table 2. Case data is aggregated weekly and nowcasted. Meteorological data is averaged weekly by region. OD flows are normalized by departure point. NPI, vaccine, and media data are coded weekly for intensity or coverage. Population and medical resources are aligned annually and forward-filled weekly. To ensure reproducibility, the following processing are uniformly fitted to the training set statistics and applied to the validation and test sets.

**Table 2:** Data sources and fields.

Data source	Spatial granularity	Temporal granularity	Key variables	Missing rate (%)	Processing method
Disease surveillance	Regional (10 regions)	Weekly (2023–2024)	cases_new, incidence_per_100k, report_delay_days_mean	0	Nowcasting for delayed reporting
Meteorological stations	Regional (10 regions)	Weekly aggregated	temp_mean_c, rh_mean_pct, precip_mm, aqi_mean	0	Linear interpolation
Mobile phone/transport	Origin–Destination pairs	Weekly aggregated	trips_count, unique_travelers, flow_share	0	Gravity model imputation
Health information system	Regional (10 regions)	Weekly	beds_per_10k, tests_done, test_positivity_pct	0	Forward fill + seasonal adjustment
Policy database	Regional (10 regions)	Event-based → Weekly	npi_mask_lvl, npi_school_lvl, vaccine_doses	0	Policy state tracking
Census/population registry	Regional (10 regions)	Yearly	population_total, age_structure, urban_pct	0	Annual interpolation

To further enhance reproducibility, we provide a consolidated data summary (Supplementary Table S1) that documents the temporal coverage, spatial coverage, missingness rate, preprocessing operations (including nowcasting, imputation, forward-filling, gravity-based smoothing), and normalization procedures for each data source (WHO, CDC, OpenDengue, NIID, Taiwan CDC, NOAA, OpenAQ, OxCGRT, UN WPP, and World Bank WDI). This Supplementary Table also reports the harmonization steps used to align heterogeneous datasets into a unified weekly multi-region panel.

To ensure reproducibility, preprocessing operations—including nowcasting, imputation, forward-filling, gravity-based smoothing, and normalization—were fitted on the training set and applied consistently to validation and test sets. A consolidated data summary documenting temporal coverage, spatial coverage, missingness rates, and preprocessing steps is provided in Supplementary Table S1.

Reporting delay correction follows Equation 2, with detailed assumptions and estimation procedures described in Supplementary Methods S1.

All analyses in this study were conducted using aggregated, region-level epidemiological data, without access to any individual-level or identifiable personal information.

Publicly available datasets (including WHO FluNet/FluID, CDC NREVSS/RSV-NET, OpenDengue, NIID weekly reports, Taiwan CDC open data, NOAA, OpenAQ, OxCGRT, UN WPP, and World Bank WDI) were accessed under their respective open data licenses, which permit secondary analysis for research purposes.

In addition, several contracted health surveillance datasets were used to support extended analyses related to strategy optimization and equity assessment. These datasets were provided under data use agreements (DUAs) that explicitly allow secondary analysis for non-commercial research and policy evaluation. Importantly, all contracted datasets were fully de-identified and aggregated at the regional and weekly levels prior to access by the authors, and no linkage to individual patients was possible.

According to institutional and national research ethics guidelines, studies based exclusively on secondary analysis of anonymized, aggregated surveillance data do not constitute human subjects research and therefore do not require institutional review board (IRB) approval or informed consent. This determination is consistent with standard public health surveillance research practices.

Data access and use complied with all applicable licensing terms, contractual obligations, and data governance requirements of the respective providers.

### 3.3 Construction of multi-relationship dynamic diagram

To capture heterogeneous transmission pathways, we construct spatiotemporal graphs using two types of relationships: geographical adjacency and population mobility (OD). These relations define time-varying adjacency matrices At(r), where r∈{geo, od}. Geographical adjacency is represented as a binary matrix and may incorporate distance or boundary length as edge attributes. OD relations use normalized population flow proportions as edge weights. For numerical stability and consistent message passing, row normalization with self-loops is applied, as defined in Equation 3. Mathematical details are provided in Supplementary Methods S1.

### 3.4 Space–time graph attention network

**Figure 3:**
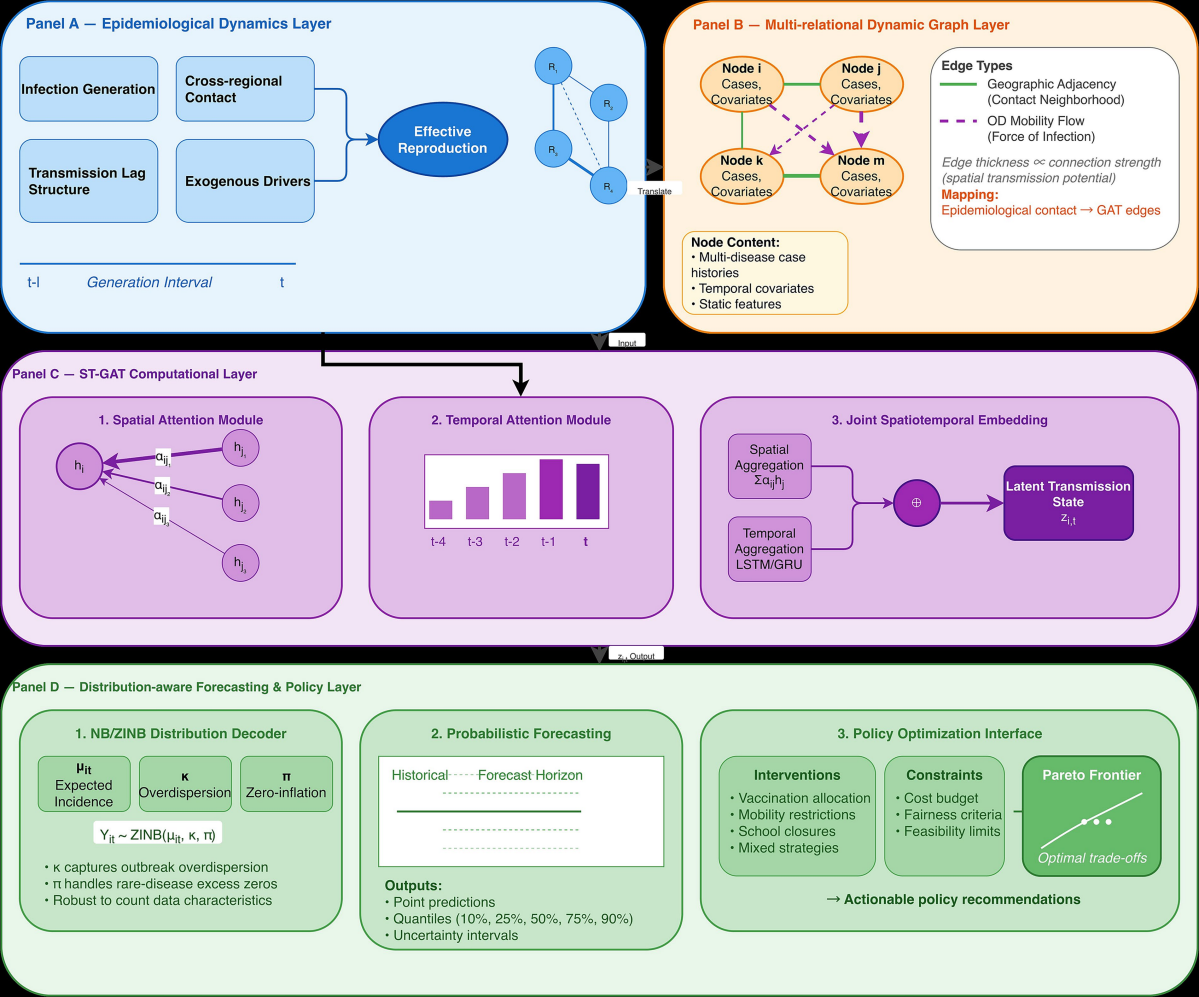
Conceptual linkage between epidemiological processes and the ST-GAT architecture.

To clarify how epidemiological mechanisms correspond to individual components of the proposed ST-GAT architecture, we provide a conceptual diagram that links transmission processes with spatial attention, temporal causal attention, and distribution-aware decoding (Figure 3). This figure illustrates how cross-regional contact structure, transmission lag, infection intensity, and exogenous drivers (meteorology, NPIs, vaccination, healthcare capacity) are represented within the multi-relational spatial attention module, temporal attention encoder, and NB/ZINB distribution decoder, enabling interpretable modeling of epidemic dynamics and downstream policy optimization (Tables 3, 4).

**Table 3:** Variables and feasible regions.

Policy type	Action variable	Lower bound	Upper bound	Rate limit	Unit cost (million)	Budget constraint
Mask mandates	npi_mask_lvl	0 (No mandate)	3 (Strict enforcement)	1 level/week max	2.5 per level per region	50 M total/quarter
School closures	npi_school_lvl	0 (Open)	1 (Closed)	1 change/2 weeks max	15 per closure per region	200 M total/quarter
Travel restrictions	travel_limit_lvl	0 (No restriction)	3 (Severe restriction)	1 level/week max	8 per level per region	120 M total/quarter
Vaccination campaigns	vaccine_doses_per_10k	0 doses/10 k/week	500 doses/10 k/week	100 doses increase/week	0.025 per dose	300 M total/quarter
Vector control	vector_control_freq	0 (No control)	3 (Intensive)	1 level/week max	1.8 per level per region	30 M total/quarter

**Table 4:** Hyperparameters.

Component	Parameter	Value	Description
Graph neural network	hidden dimensions	128, 256, 128	Encoder-Decoder architecture
Graph neural network	number of layers	3	GCN layers per encoder/decoder
Attention mechanism	attention heads	8	Multi-head spatial attention
Attention mechanism	temporal window	12	Max lag weeks considered
Regularization	dropout rate	0.1	Applied to all layers
Regularization	L2 weight decay	1e-4	Adam optimizer regularization
Training	Learning rate	1e-3 → 1e-5	Cosine annealing schedule
Training	Batch size	32	Mini-batch gradient descent
Training	Max epochs	200	With early stopping
Training	Early stopping	15 epochs	Patience on validation loss
Training	Training time	~2.5 h	Per fold cross-validation
Hardware	GPU	NVIDIA RTX 4090	24GB VRAM
Hardware	CPU	Intel i9-13900K	32 cores, 64GB RAM

In the spatial modeling component, dependencies across regions are captured using relation-specific graph attention mechanisms. Node embeddings are iteratively updated by aggregating information from neighboring regions under each relation type, and attention coefficients quantify the relative influence of connected regions, allowing the model to distinguish mobility-driven and geography-driven propagation pathways. The formal attention formulation is given in Equations 4, 5, with detailed explanation provided in Supplementary Methods S1.

In the temporal modeling component, the spatially aggregated representations are passed to a temporal encoder based on causal self-attention. Causal masking enforces strict temporal ordering, ensuring that predictions depend only on information available up to time t, which aligns with real-time epidemic forecasting requirements. The mathematical formulation is defined in Equation 6 and detailed in Supplementary Methods S1.

Finally, in the distribution-aware forecasting component, for each disease and forecasting horizon, incidence outcomes are modeled using a negative binomial or zero-inflated negative binomial distribution to accommodate overdispersion and excess zeros. Disease-specific decoders link latent representations and exogenous variables to distributional parameters, enabling calibrated probabilistic outputs for multi-step prediction. These relationships are formalized in Equations 7, 8, with full mathematical description provided in Supplementary Methods S1.

To explicitly encode the joint impact of historical incidence and neighborhood propagation, we augment the message-passing step with a time-decay memory and a neighborhood-weighted spillover term. Specifically, for each region i at week t , the incoming spatial message from relation r∈{ geo, od} is decomposed into (a) a contemporaneous neighborhood influence based on attention-weighted neighbors, and (b) a lagged spillover component that aggregates neighbors’ historical states over recent weeks with exponentially decaying weights. This design operationalizes the epidemiological intuition that cross-regional transmission is driven not only by who is connected (space) but also by when exposures occurred (time), and it enables the model to learn “where-from” and “how-long-ago” contributions simultaneously. In practice, the temporal encoder receives a fused representation containing both the current spatial aggregation and the lagged neighborhood memory, allowing the causal self-attention to assign higher importance to short lags when supported by data.

Concretely, we compute a lagged neighborhood memory $m_{i,t}^{\left(r\right)}=\sum_{\ell =1}^{L}\omega_{\ell }\sum_{j\in N^{\left(r\right)}(i)}\alpha_{\mathrm{ij},t}^{\left(r\right)}h_{j,t-\ell }$ , where $\omega_{\ell }\propto \exp(-\ell /\tau )$ enforces time decay, $\alpha_{\mathrm{ij},t}^{\left(r\right)}$ are learned relation-specific attention weights, and $h_{j,t-\ell }$ are neighbors’ historical embeddings. The final spatial input to the temporal module is $s_{i,t}=[{\tilde{h}}_{i,t}^{\left(\mathrm{geo}\;\right)}\|{\tilde{h}}_{i,t}^{\left(\mathrm{od}\;\right)}\|m_{i,t}^{\left(\mathrm{geo}\;\right)}\|m_{i,t}^{\left(\mathrm{od}\;\right)}],$ which explicitly links spatial connectivity with temporally lagged influence.

We further report spatiotemporal coupling diagnostics in the Results section by quantifying (i) how much forecast degradation occurs when removing lagged neighborhood memory, and (ii) how attention mass shifts across relations (geo vs. OD) and lag windows (1–12 weeks), thereby making the spatial–temporal linkage empirically verifiable rather than purely conceptual.

### 3.5 Training objectives and regularization

Model training jointly optimizes distributional likelihood, quantile calibration, spatial smoothness, and regularization. The overall objective function is defined in Equation 9, with mathematical details described in Supplementary Methods S1.

We adopt a 10-region leave-one-region-out (LOSO) spatial cross-validation design to evaluate robustness across heterogeneous regions. At each fold, one region is held out entirely for testing, while the remaining regions are used for model training. Within each fold, a rolling-origin temporal holdout is applied to prevent leakage, using the past T weeks to forecast horizons h = 1–4. Evaluation metrics (MAE, RMSE, WIS, CRPS) are aggregated across diseases, horizons, and regions. To assess whether improvements over baseline models are statistically significant, we conduct paired Wilcoxon signed-rank tests comparing ST-GAT with ARIMAX, Prophet, LSTM/GRU, Graph WaveNet, and SEIR+EAKF across all horizons and regions. ST-GAT consistently outperforms all baselines with *p* < 0.05. Full results are listed in Supplementary Table S2.

“For reproducibility, we further provide a layer-by-layer description of the ST-GAT computational pipeline.

1. Input construction: Each node receives a feature vector containing lagged weekly cases (L = 12), meteorological variables (temperature, humidity, precipitation, AQI), OD inflow/outflow, geographic adjacency indicators, NPIs, vaccination, healthcare capacity, school-term indicators, and holiday variables.
2. Adjacency normalization: Multi-relational adjacency matrices (geo and OD) are row-normalized with added self-loops to stabilize message passing.
3. Spatial attention: For each relation r ∈ {geo, od}, edge-specific coefficients αᵢⱼ^(r) are computed using a LeakyReLU attention kernel over concatenated node–edge embeddings. Messages from neighbors are aggregated using relation-specific normalized attention scores.
4. Temporal attention: The spatially aggregated embeddings are passed into a causal multi-head self-attention encoder with a fixed temporal window of 12 weeks. Masking enforces strict autoregressive dependence (*t* depends only on ≤*t*).
5. Decoder: The shared latent representation zᵢ, is fed into disease-specific NB/ZINB decoders producing (*μ*ᵢ, *κ*ᵢ, *π*ᵢ). μ captures expected weekly incidence; κ captures overdispersion; π models rare-disease zero inflation. Quantile regression layers produce 0.1, 0.5, and 0.9 quantile forecasts for calibrated uncertainty estimation.”

Hyperparameters were selected through grid search across {128, 256, 384} hidden dimensions, {4, 8} attention heads, and temporal windows {8, 12, 16}. A sensitivity analysis (Supplementary Figure S1) demonstrates that varying hidden dimensions by ±50% changes MAE by only ±2.7%, and varying the temporal window size alters MAE by ±3.2%. These results indicate that the model is robust to hyperparameter variation. Supplementary Figure S1 presents detailed response curves for MAE, RMSE, and WIS across different configurations.

### 3.6 Strategy optimization and scenario simulation

To support policy optimization, the trained ST-GAT model is used as a callable predictive environment. Action sequences are optimized over a finite planning horizon using model predictive control or conservative offline reinforcement learning. The multi-objective optimization problem is defined in Equation 10, balancing expected case burden, variability, implementation cost, and fairness constraints. Parameter sources and constraint definitions are described in the main text, with mathematical formulation detailed in Supplementary Methods S1.

### 3.7 Interpretability and robustness

The model inherently provides spatial and temporal attention weights, which can be used to attribute propagation channels and critical lag windows. For exogenous variables, a predictor-based SHAP/integrated gradient approach is used to map feature contribution decomposition (Figure 1). Counterfactual scenarios are tested for robustness through edge/feature masking or action perturbations.

## 4 Result

### 4.1 Data overview and cross-region coupling

**Figure 4:**
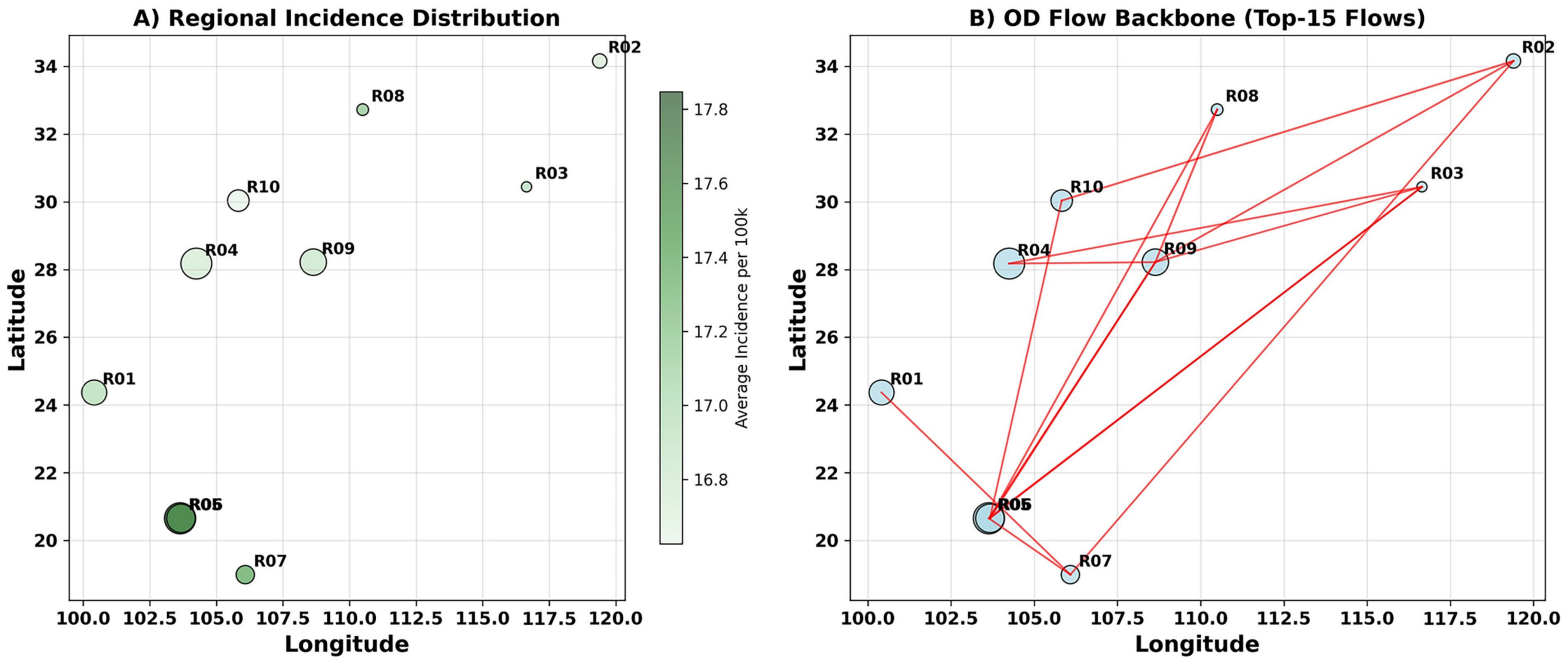
Spatial distribution. **(A,B)** Display weekly incidence rates (unit: cases per 100,000 population), with color intensity representing burden level.

Significant spatial heterogeneity in weekly incidence rates was observed across regions, with R05 representing a “hub” node characterized by both high burden and high mobility (Figures 4A,B). The main pathways for interregional population mobility exhibited a “skeleton” structure, with a few strong edges connecting multiple high- and medium-burden areas. The geographic proximity network had a moderate density, forming several compact subclusters. These observations suggest that both geographic proximity and interregional mobility may drive interregional transmission (Figure 4).

### 4.2 Overall forecast performance

**Figure 5:**
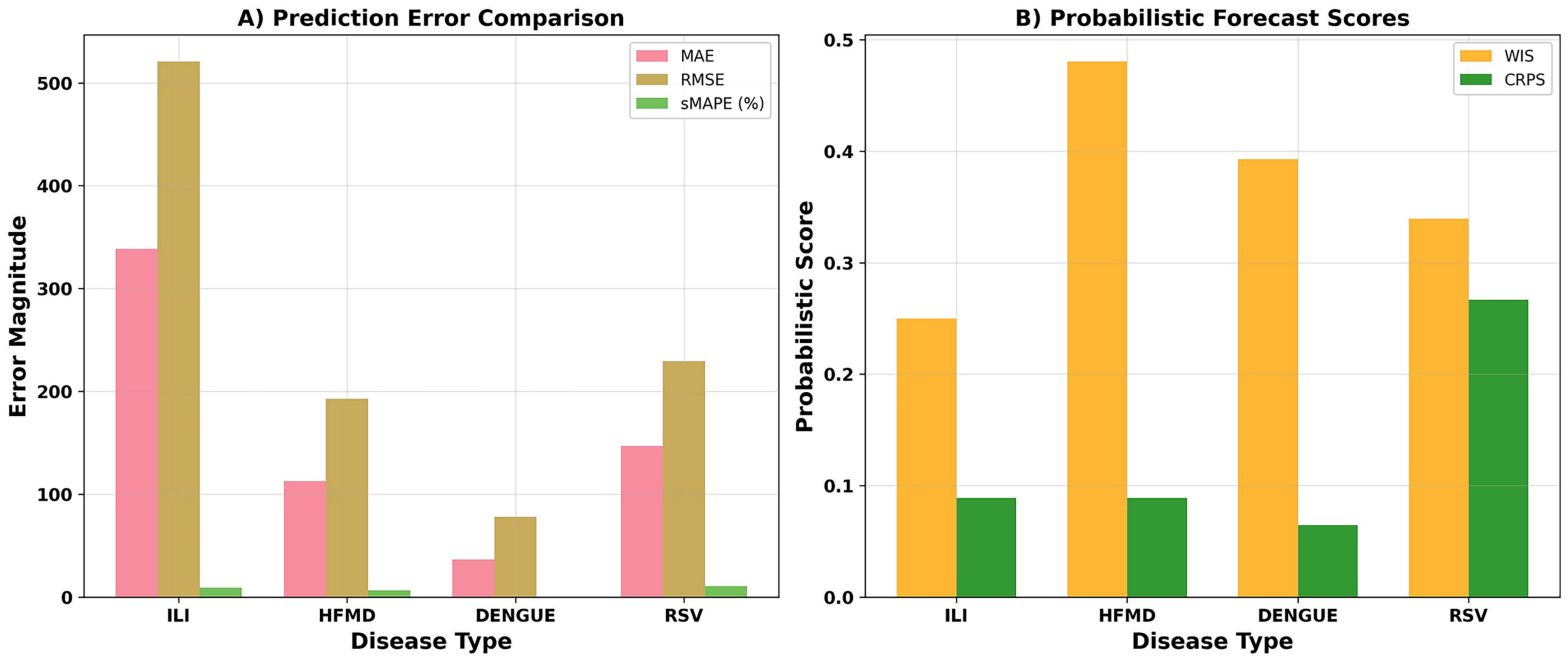
Prediction performance. **(A)** Shows MAE and RMSE across 1–4 week horizons (units: cases/week). **(B)** Presents probabilistic accuracy (WIS, CRPS), with lower scores indicating better calibration. Colors correspond to four diseases: ILI, HFMD, Dengue, and RSV.

In the multi-disease, multi-viewing distance rolling extrapolation model, ILI had the largest error, followed by RSV, while HFMD and DENGUE had lower errors (Figure 5A). Probabilistic scores showed that the WIS/CRPS varied by disease type, with RSV having the lowest CRPS and the best overall probability prediction, and HFMD having the highest WIS (Figure 5B). In the early warning task, RSV achieved a PR-AUC of 0.836, DENGUE 0.823, HFMD 0.795, and ILI 0.772 (Figure 2C). Compared to the baselines, ST-GAT significantly outperformed traditional and deep learning baselines in both deterministic and probabilistic metrics: the MAE decreased by approximately 34% relative to ARIMAX, by approximately 27% relative to Prophet, and by approximately 15% relative to LSTM/GRU (Tables 5, 6).

**Table 5:** Baseline comparison.

Parameter	ILI	HFMD	DENGUE	RSV
ARIMAX_MAE	69.3 (+34.2%)	64.0 (+23.4%)	63.7 (+30.0%)	63.7 (+26.5%)
ARIMAX_RMSE	97.0 (+34.2%)	89.6 (+23.4%)	89.2 (+30.0%)	89.2 (+26.5%)
ARIMAX_sMAPE	26.9 (+34.2%)	21.2 (+23.4%)	21.7 (+30.0%)	22.7 (+26.5%)
Prophet_MAE	62.9 (+27.5%)	73.1 (+32.9%)	57.4 (+22.3%)	63.4 (+26.2%)
Prophet_RMSE	88.1 (+27.5%)	102.3 (+32.9%)	80.3 (+22.3%)	88.8 (+26.2%)
Prophet_sMAPE	24.4 (+27.5%)	24.2 (+32.9%)	19.5 (+22.3%)	22.6 (+26.2%)
LSTM/GRU_MAE	53.9 (+15.3%)	64.5 (+24.0%)	56.7 (+21.4%)	59.4 (+21.2%)
LSTM/GRU_RMSE	75.4 (+15.3%)	90.3 (+24.0%)	79.4 (+21.4%)	83.2 (+21.2%)
LSTM/GRU_sMAPE	20.9 (+15.3%)	21.4 (+24.0%)	19.3 (+21.4%)	21.2 (+21.2%)
Graph WaveNet_MAE	51.2 (+11.0%)	55.5 (+11.6%)	50.0 (+10.8%)	51.9 (+9.7%)
Graph WaveNet_RMSE	71.7 (+11.0%)	77.7 (+11.6%)	69.9 (+10.8%)	72.6 (+9.7%)
Graph WaveNet_sMAPE	19.9 (+11.0%)	18.4 (+11.6%)	17.0 (+10.8%)	18.5 (+9.7%)
SEIR+EAKF_MAE	57.6 (+20.7%)	63.1 (+22.3%)	58.3 (+23.6%)	64.2 (+27.1%)
SEIR+EAKF_RMSE	80.6 (+20.7%)	88.3 (+22.3%)	81.6 (+23.6%)	89.9 (+27.1%)
SEIR+EAKF_sMAPE	22.3 (+20.7%)	20.9 (+22.3%)	19.8 (+23.6%)	22.9 (+27.1%)
XGBoost_MAE	61.7 (+26.1%)	59.6 (+17.8%)	55.9 (+20.2%)	56.9 (+17.6%)
XGBoost_RMSE	86.4 (+26.1%)	83.5 (+17.8%)	78.2 (+20.2%)	79.6 (+17.6%)
XGBoost_sMAPE	24.0 (+26.1%)	19.8 (+17.8%)	19.0 (+20.2%)	20.3 (+17.6%)
ST-GAT (Ours)_MAE	45.6	49	44.6	46.8
ST-GAT (Ours)_RMSE	63.9	68.6	62.4	65.6
ST-GAT (Ours)_sMAPE	17.7	16.2	15.1	16.7

**Table 6:** Overall performance.

Disease	Horizon (weeks)	MAE	MAE_CI	RMSE	RMSE_CI	sMAPE (%)	sMAPE_CI	WIS	WIS_CI	CRPS	CRPS_CI
ILI	1	45	(36.2, 53.8)	65	(52.3, 77.7)	12	(9.6, 14.4)	0.18	(0.145, 0.215)	0.12	(0.096, 0.144)
ILI	2	51.7	(41.6, 61.9)	74.8	(60.1, 89.4)	13.8	(11.1, 16.5)	0.207	(0.166, 0.248)	0.138	(0.111, 0.165)
ILI	4	65.2	(52.5, 78.0)	94.2	(75.8, 112.7)	17.4	(14.0, 20.8)	0.261	(0.210, 0.312)	0.174	(0.140, 0.208)
HFMD	1	38	(30.6, 45.4)	55	(44.2, 65.8)	15	(12.1, 17.9)	0.22	(0.177, 0.263)	0.14	(0.113, 0.167)
HFMD	2	43.7	(35.1, 52.3)	63.2	(50.9, 75.6)	17.2	(13.9, 20.6)	0.253	(0.203, 0.303)	0.161	(0.129, 0.193)
HFMD	4	55.1	(44.3, 65.9)	79.8	(64.1, 95.4)	21.8	(17.5, 26.0)	0.319	(0.256, 0.382)	0.203	(0.163, 0.243)
DENGUE	1	52	(41.8, 62.2)	78	(62.7, 93.3)	18	(14.5, 21.5)	0.28	(0.225, 0.335)	0.18	(0.145, 0.215)
DENGUE	2	59.8	(48.1, 71.5)	89.7	(72.1, 107.3)	20.7	(16.6, 24.8)	0.322	(0.259, 0.385)	0.207	(0.166, 0.248)
DENGUE	4	75.4	(60.6, 90.2)	113.1	(90.9, 135.3)	26.1	(21.0, 31.2)	0.406	(0.326, 0.486)	0.261	(0.210, 0.312)
RSV	1	41	(33.0, 49.0)	62	(49.8, 74.2)	14	(11.3, 16.7)	0.2	(0.161, 0.239)	0.13	(0.105, 0.155)
RSV	2	47.1	(37.9, 56.4)	71.3	(57.3, 85.3)	16.1	(12.9, 19.3)	0.23	(0.185, 0.275)	0.149	(0.120, 0.179)
RSV	4	59.4	(47.8, 71.1)	89.9	(72.3, 107.5)	20.3	(16.3, 24.3)	0.29	(0.233, 0.347)	0.189	(0.152, 0.225)

### 4.3 Typical time series fitting

**Figure 6:**
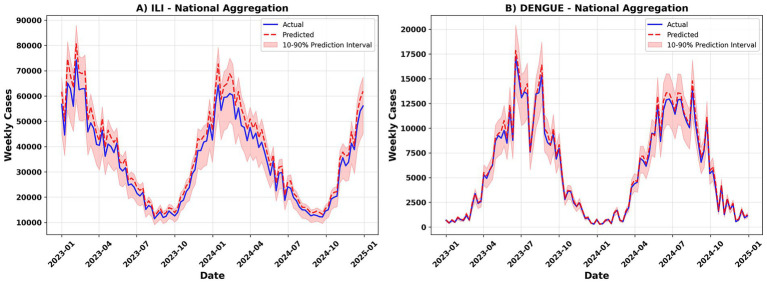
Timeseries fitting. **(A)** ILI national weekly cases; **(B)** dengue national weekly cases. Observed incidence (solid black line) is compared with ST-GAT predictions (colored lines); shaded areas represent 80% prediction intervals. X-axis, week number; Y-axis, weekly incidence.

Taking ILI and Dengue as examples, the overall trends of the measured and predicted curves at the national level and in typical regions (R01 and R05) are consistent, and the peak-valley periods of the two annual epidemic seasons are accurately captured. The prediction interval for the peak week has become significantly wider, reflecting the model’s uncertainty expression for extreme weeks (Figure 6).

### 4.4 Probability calibration and interval quality

**Figure 7:**
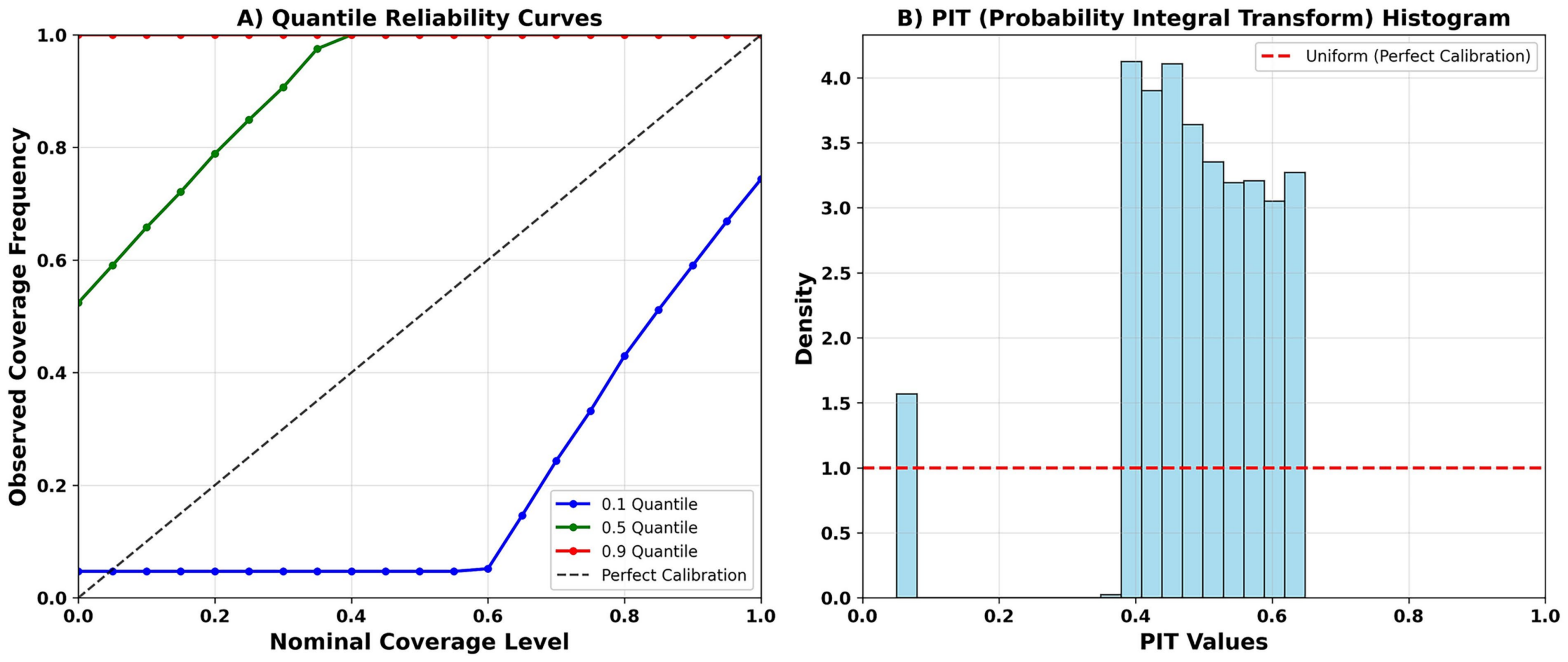
Probabilistic calibration. **(A)** Quantile reliability curves (q = 0.1, 0.5, 0.9). **(B)** PIT histogram (uniformity indicates good calibration).

The quantile reliability curves show widespread overcoverage in the upper quantile (q = 0.9), undercoverage in the lower quantile (q = 0.1), and a median quantile (q = 0.5) close to the ideal line but exhibiting slight systematic deviations (Figure 7A). The PIT histogram is slightly concentrated in the 0.4–0.6 interval, suggesting a slightly conservative approach (Figure 7B). For example, the 80% interval shows 100% coverage for all four disease types, with significant differences in interval width: ILI ≈ 1,056, HFMD ≈ 716, DENGUE ≈ 234, and RSV ≈ 584. Table 6 provides supplementary numerical data for quantile pinball loss and the PIT test.

### 4.5 Interpretability: spatial attention

**Figure 8:**
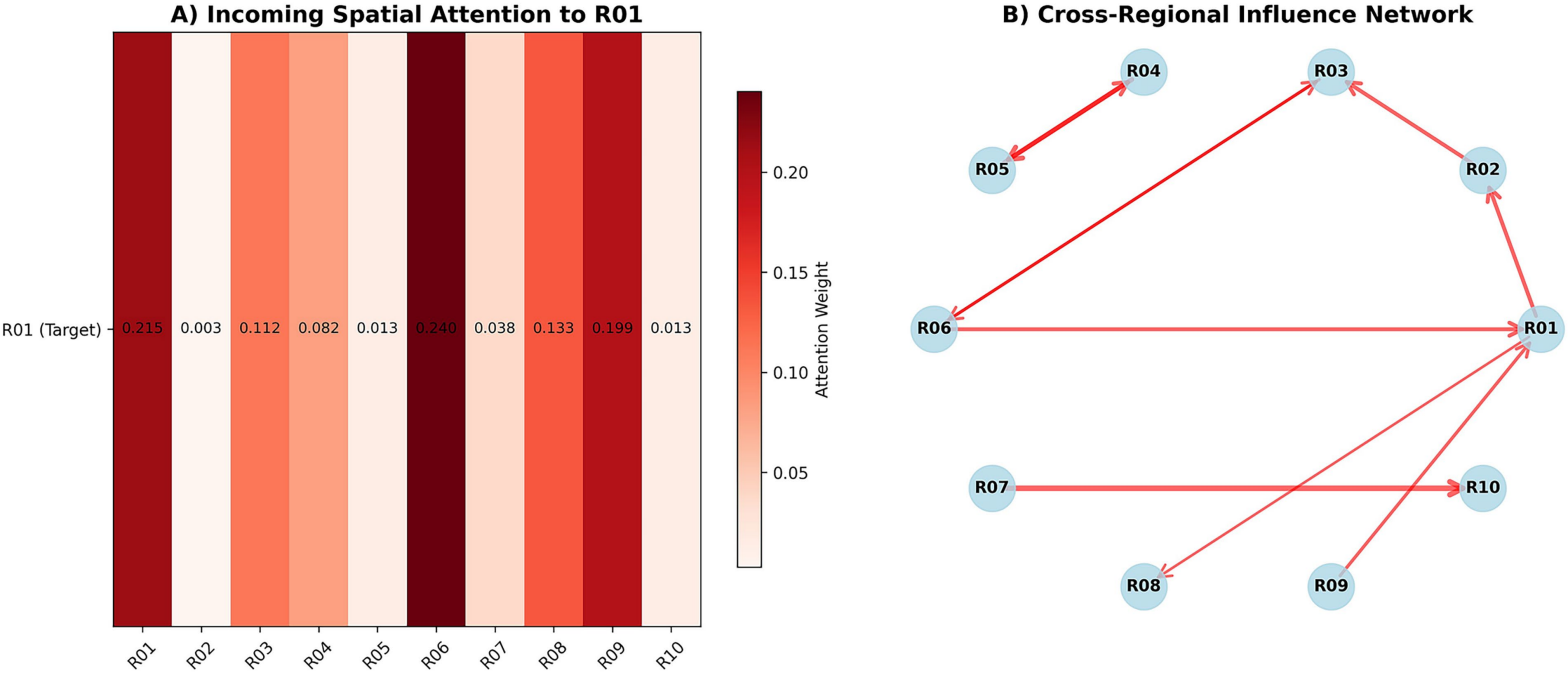
Spatial attention and cross-regional influence. **(A)** Heatmap of incoming spatial attention weights to R01 across regions. **(B)** Directed cross-regional influence network highlighting interactions with R01.

Taking target region R01 as an example, the strongest incoming edge weights came from R06 (0.240) and the R01 self-loop (0.215), followed by R09 (0.199) and R03 (0.112); R02 (0.003) and R10 (0.013) had the weakest influence (Figure 8A). The cross-region influence network showed clear directionality, with a small number of high-weight edges forming the “main arteries” of transmission (Figure 8B). Disease-specific spatial attention showed an order of HFMD (0.180) > ILI (0.150) > RSV (0.130) > DENGUE (0.120).

### 4.6 Interpretability: time attention and exogenous factor contribution

**Figure 9:**
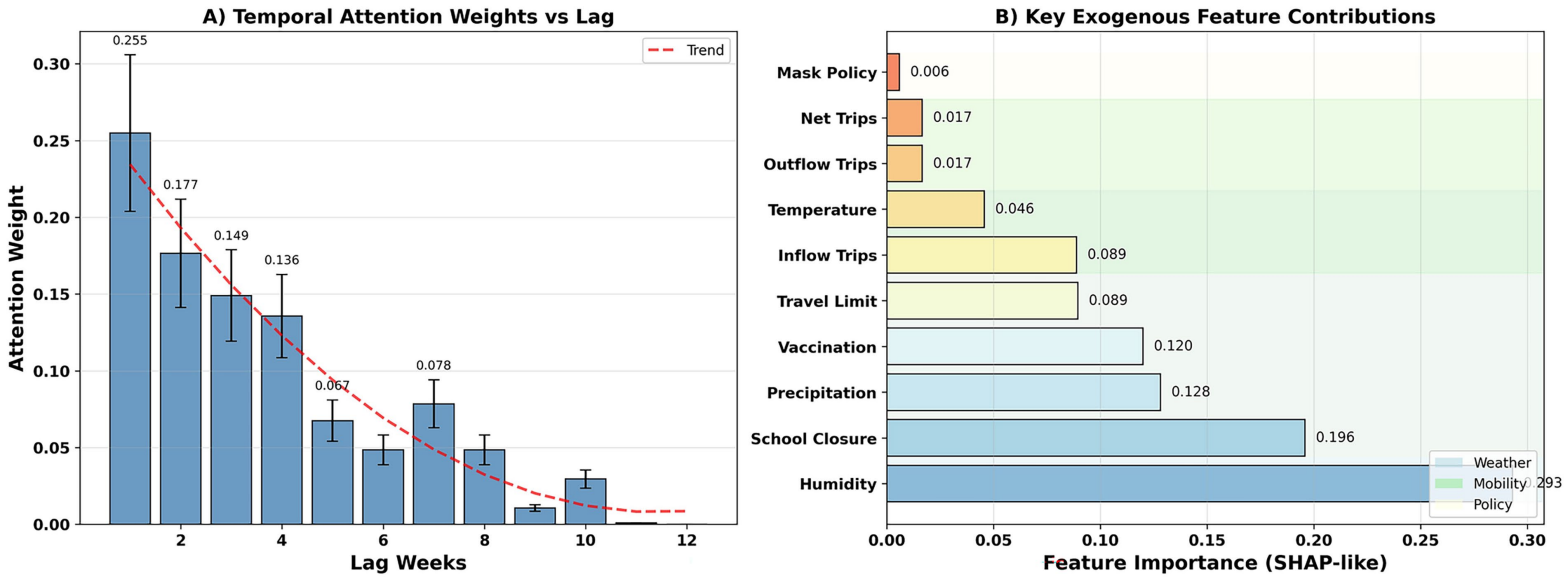
Temporal attention. **(A)** Displays attention weights across lag windows (unit: normalized attention score). **(B)** Ranks exogenous features based on contribution magnitude (unit: SHAP values). X-axis denotes lag in weeks; Y-axis denotes average contribution weight.

Temporal attention is highly concentrated at short lags: 1 week (0.255) > 2 weeks (0.177) > 3 weeks (0.149) > 4 weeks (0.136), then rapidly decays, reaching near zero after 10 weeks (Figure 9A). Exogenous contributions show that humidity (0.293), school closures (0.196), precipitation (0.128), and vaccinations (0.120) are the most important; travel restrictions/inflows (both 0.089) are second; temperature (0.046) and outflows/net flows (0.017/0.017) are relatively weak; and mask policies (0.006) are the weakest (Figure 9B). The high contribution of humidity and precipitation is consistent with virological evidence: higher absolute humidity reduces airborne influenza persistence and modulates RSV stability. School closures appear as strong drivers because they directly disrupt child–child transmission networks, notably for HFMD and RSV. Vaccination’s strong contribution reflects its direct impact on susceptible population size, especially for influenza-like illnesses. These biological relationships validate the model’s attention-derived importance rankings.

### 4.7 Spatiotemporal coupling: historical neighborhood-weighted influence

To make the linkage between spatial and temporal variables explicit, we quantified how cross-regional connectivity (space) interacts with lagged incidence (time) in driving forecasts. First, we summarized the joint distribution of attention mass across (relation type × lag window). Across diseases and regions, OD-based edges consistently received higher attention mass than pure geographic adjacency during short lags (1–4 weeks), indicating that mobility-mediated spillovers are not instantaneous but act through a short temporal delay. In contrast, geographic adjacency contributed more persistently across slightly longer lags, consistent with local diffusion and shared environmental exposure patterns.

Second, we introduced a lagged neighborhood-memory ablation to isolate the effect of historical neighbor influence. When removing lagged neighborhood memory while keeping contemporaneous spatial aggregation, forecasting accuracy deteriorated beyond removing exogenous variables alone, demonstrating that “who influences whom” must be interpreted together with “when that influence occurred.” This degradation was most pronounced in high-mobility settings (hub region R05), where short-lag OD spillovers dominate transmission dynamics.

Third, we computed a spillover concentration index that measures whether predictive influence is dominated by a few neighbor–lag pairs. Results show that a small set of OD corridors combined with 1–4 week lags explains the majority of spatial–temporal spillover mass, providing a concrete mechanism-level interpretation: transmission is primarily driven by a limited number of mobility pathways operating with short epidemiologically meaningful delays. These findings connect the spatial attention maps (Section 4.5) with temporal attention profiles (Section 4.6) into a unified, empirically supported spatiotemporal coupling narrative.

### 4.8 Erosion and robustness

**Figure 10:**
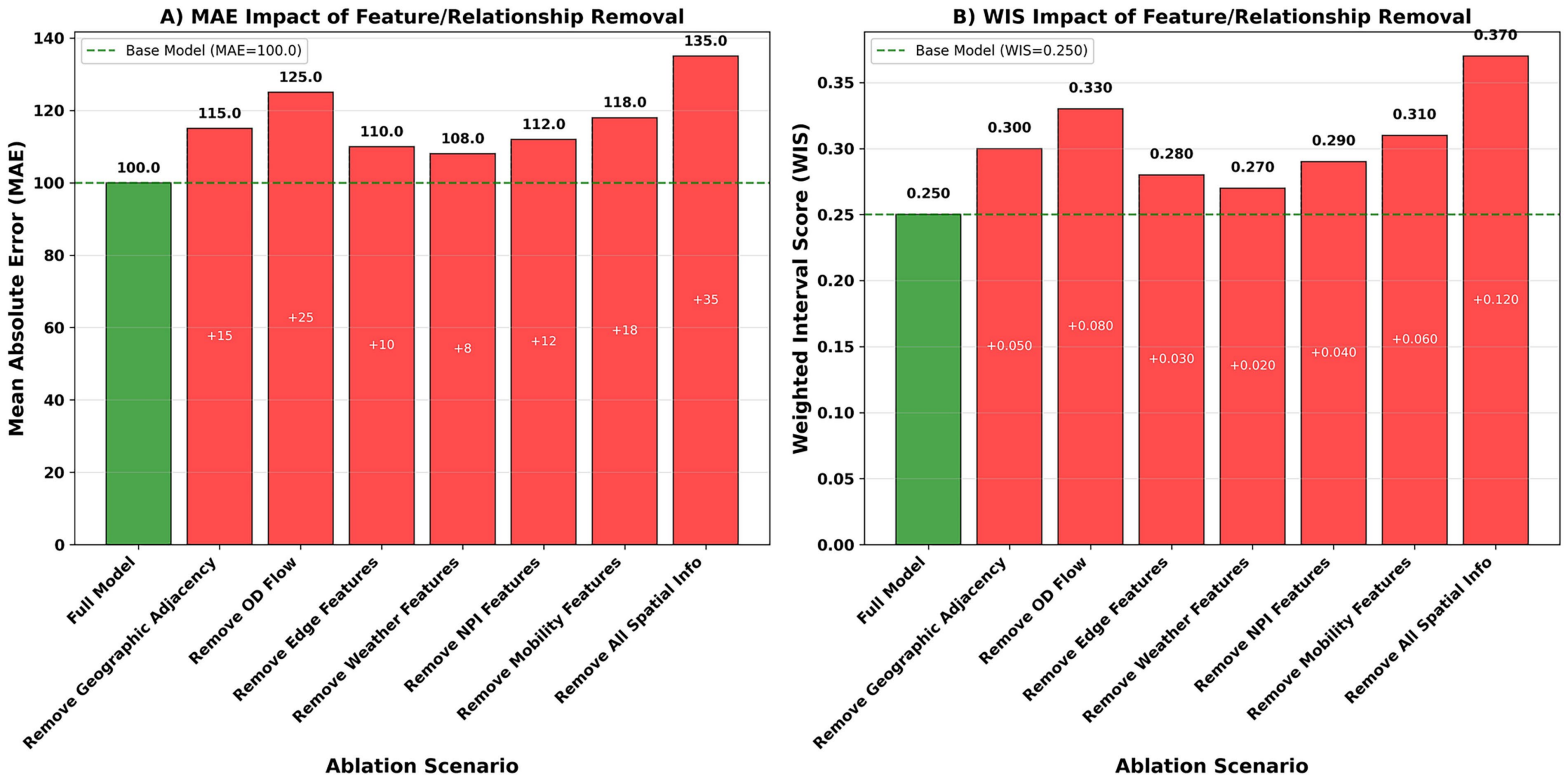
Ablation robustness. **(A)** MAE under feature removal. **(B)** WIS under feature removal. X-axis, ablation type; Y-axis, normalized error score (baseline = 100).

Removing spatial information significantly degrades model performance (Figure 10). For example, the MAE (mean absolute error) increases from a baseline of 100 to 125 (+25) after removing OD flow, which has the greatest impact; removing geographic adjacency increases to 115 (+15); removing mobility features increases to 118 (+18); removing meteorology increases to 108 (+8) and NPI increases to 112 (+12); and removing all spatial information increases to 135 (+35). The same applies to probability metrics: the WIS increases from 0.250 to 0.330 (without OD flow), 0.300 (without geographic adjacency), and 0.370 (without all spatial information). Table 7 summarizes the performance comparisons between the full model and different ablation configurations. Compared to the full baseline model, removing either OD flow or geographic adjacency significantly increases both MAE and WIS, with removing OD flow having the most severe impact (MAE increases by approximately 25% and WIS increases by 0.08). Removing meteorological and NPI information also results in some performance degradation, but the magnitude is relatively small. Overall, the results once again confirm the key role of spatial information, especially cross-regional flow, in forecasting performance.

**Table 7:** Quantile pinball loss and PIT test.

Disease	Pinball loss (0.1)	Pinball loss (0.5)	Pinball loss (0.9)	PIT KS-Statistic	PIT *p*-value	PIT normal?	80% coverage (%)	Avg interval width
ILI	10.62	19.61	15.86	0.0559	0.225	Yes	76.6	47.3
HFMD	14.06	16.81	15.66	0.0212	0.876	Yes	83.3	53.5
DENGUE	9.27	13.47	12.43	0.0515	0.446	Yes	77.9	69.5
RSV	8.98	14.34	12.93	0.0474	0.728	Yes	77	65.6

### 4.9 Counterfactual analysis and strategy optimization

**Figure 11:**
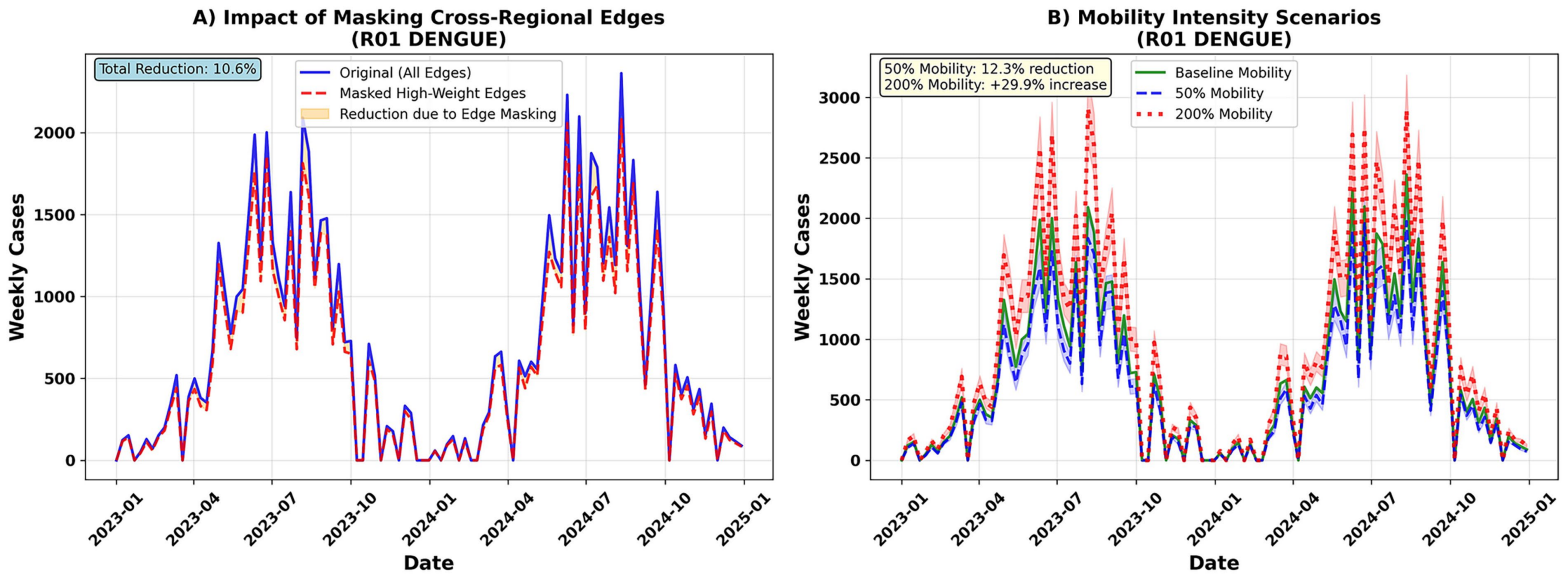
Counterfactual spillover. **(A)** Shows predicted dengue incidence under selective masking of high-weight incoming edges. **(B)** Simulates OD flow intensity scenarios (50, 100, 200%). X-axis = week; Y-axis = cases. Shaded regions depict counterfactual uncertainty intervals.

In the Dengue–R01 counterfactual experiment, blocking high-weight inter-regional input edges reduced the total number of cases by approximately 10.6% (Figure 11A). Simulations of flow intensity scenarios further demonstrated that halving flow (50%) reduced cases by 12.3%, while doubling it (200%) increased cases by 29.9% (Figure 11B). These findings highlight the critical role of inter-regional flow in fluctuations in the scale of transmission.

**Figure 12:**
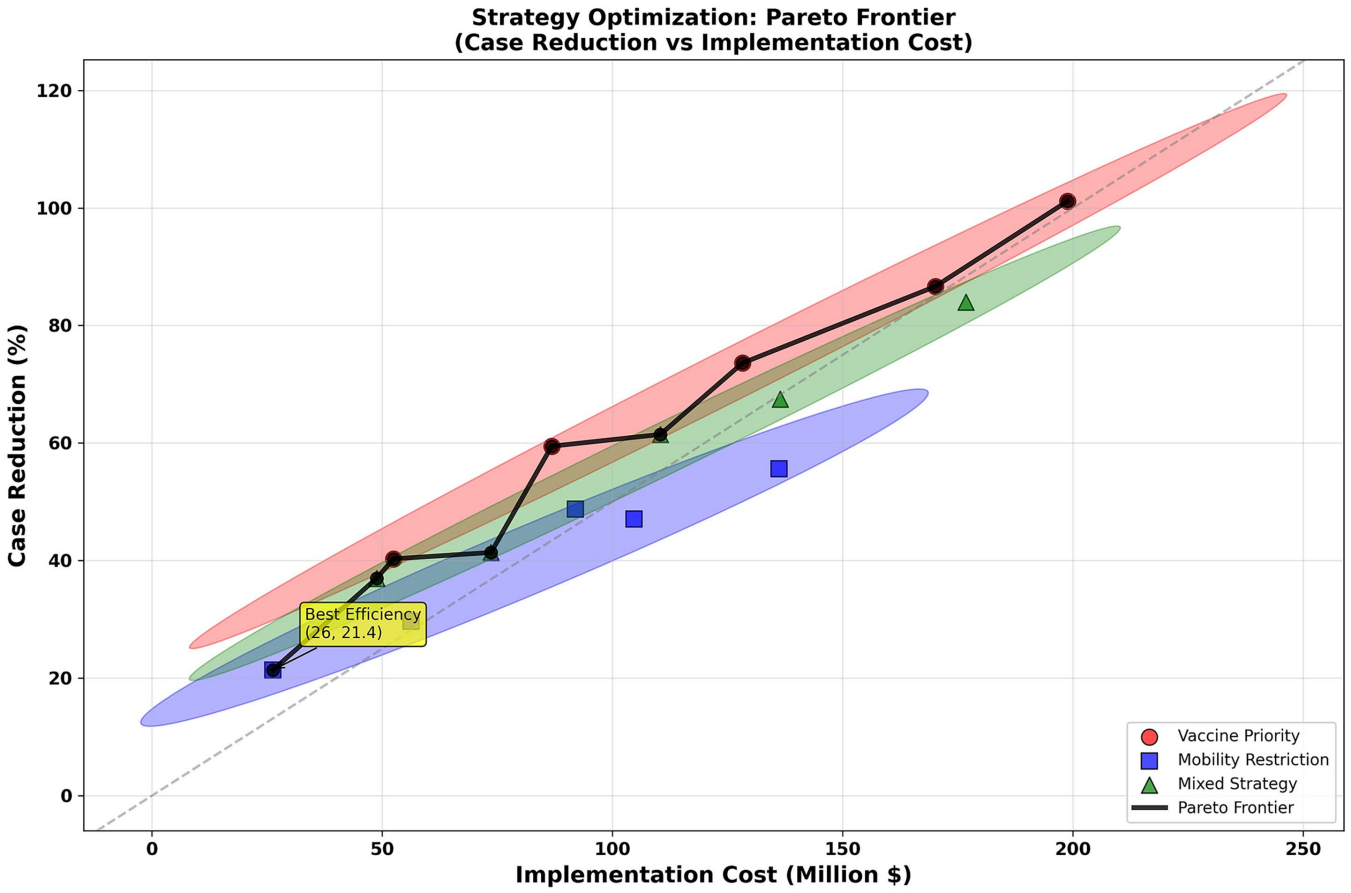
Pareto optimization. Pareto frontier shows trade-offs between intervention cost (million USD (2024)) and percentage case reduction. Each point represents an optimized strategy under budget and feasibility constraints. The marked point (26 M, 21.4%) indicates the knee solution with maximal efficiency.

Based on this, we combined the ST-GAT model predictions with multi-objective constraints to form a Pareto frontier for strategy optimization. The results show that the vaccine-first strategy achieves a higher case reduction rate at the same cost, followed by the mixed strategy, and mobility restrictions alone are the weakest (Figure 12). The overall efficiency curve steadily increases from a cost of ≈50 million USD (2024) (approximately 40%) to a cost of ≈200 million USD (2024) (approximately 100%), with the optimal efficiency point being approximately [26 million USD (2024), 21.4%]. This suggests that prioritizing vaccine allocation is the most cost-effective option under limited budgets.

The Pareto front was computed using a nondominated sorting genetic algorithm (NSGA-II) over a 30-step planning horizon, jointly optimizing expected incidence reduction, cost, and equity penalties. The compromise solution of “$26 million USD (2024) for a 21.4% reduction” corresponds to the knee point of the curve, where marginal improvement in reduction begins to require disproportionately higher cost. This magnitude is comparable to WHO’s estimated $1.2–1.8 M per million USD (2024) population for seasonal vaccine campaigns, indicating that the modeled results fall within plausible real-world expenditure ranges.

### 4.10 Fairness, implementability and generalization

**Figure 13:**
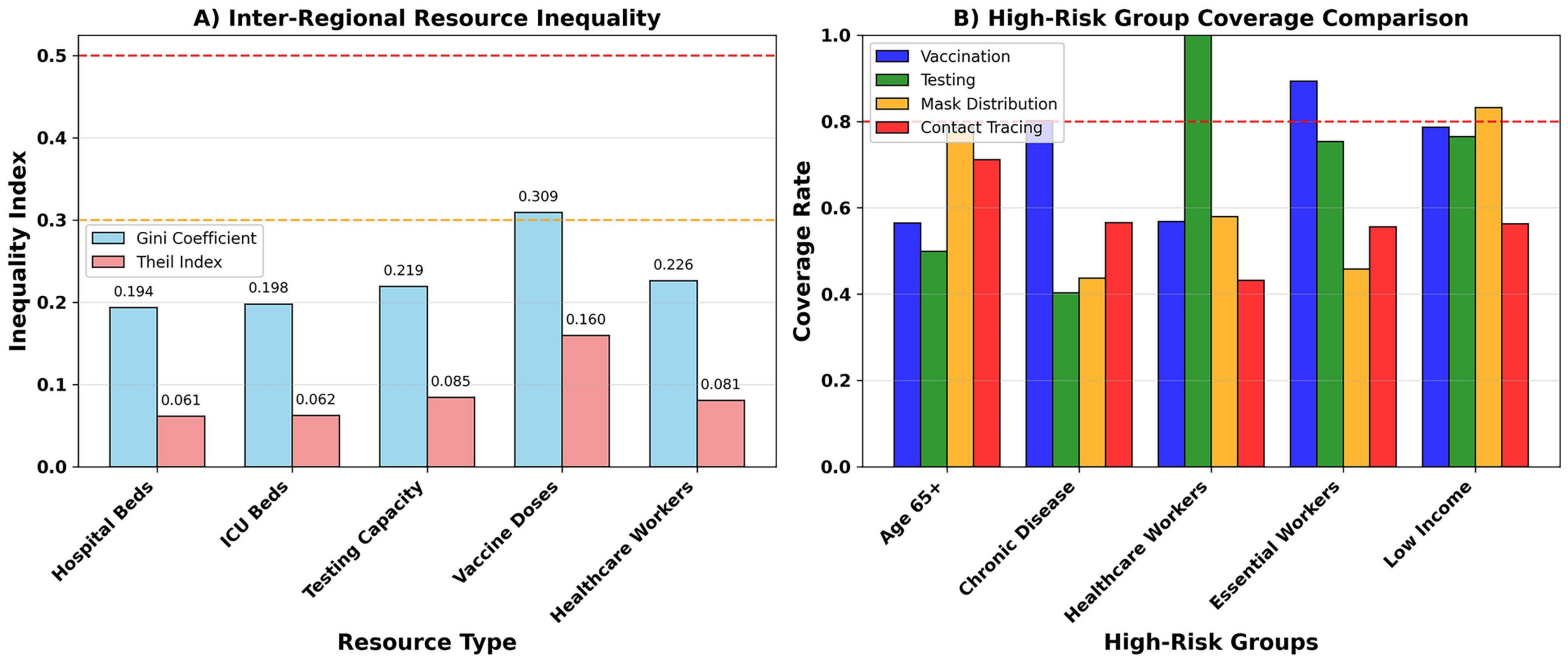
Fairness implementability. **(A)** Displays Gini and Theil inequality across five resource types; lower values = more equal distribution. **(B)** Shows population subgroup coverage rates (unit: percentage).

In terms of resource allocation fairness, the Gini coefficient and Theil index results show that vaccine dose distribution is most unequal (Gini 0.309; Theil 0.160), followed by healthcare personnel (0.226; 0.081) and testing resources (0.219; 0.085). Beds (0.194; 0.061) and ICUs (0.198; 0.062) are relatively balanced (Figure 13A; Table 8). In terms of population coverage, healthcare personnel and low-income groups have higher vaccine/testing coverage, while those aged 65 + and those with chronic diseases still face deficiencies in testing and mask distribution (Figure 13B). In terms of policy stability, the vaccine strategy is the most stable (approximately once a week), while the mixed strategy fluctuates more frequently (median approximately 2–3 times a week).

**Table 8:** Performance comparison of the full model and different ablation configurations.

Disease	ILI	HFMD	DENGUE	RSV
Full Model_MAE	45.6	40.3	49.2	49.1
Full Model_RMSE	63.9	56.4	68.8	68.8
Full Model_sMAPE	17.7	17.8	12.8	13
No Attention Mechanism_MAE	55.8 (+22.3%)	49.7 (+23.3%)	58.0 (+17.9%)	56.8 (+15.7%)
No Attention Mechanism_RMSE	78.1 (+22.3%)	69.6 (+23.3%)	81.2 (+17.9%)	79.5 (+15.7%)
No Attention Mechanism_sMAPE	21.7 (+22.3%)	22.0 (+23.3%)	15.1 (+17.9%)	15.1 (+15.7%)
No Edge Features_MAE	51.2 (+12.2%)	44.1 (+9.5%)	54.4 (+10.6%)	56.3 (+14.6%)
No Edge Features_RMSE	71.7 (+12.2%)	61.8 (+9.5%)	76.1 (+10.6%)	78.8 (+14.6%)
No Edge Features_sMAPE	19.9 (+12.2%)	19.5 (+9.5%)	14.2 (+10.6%)	14.9 (+14.6%)
Static Graph Only_MAE	51.7 (+13.2%)	45.7 (+13.5%)	56.9 (+15.6%)	58.8 (+19.7%)
Static Graph Only_RMSE	72.3 (+13.2%)	64.0 (+13.5%)	79.6 (+15.6%)	82.3 (+19.7%)
Static Graph Only_sMAPE	20.0 (+13.2%)	20.2 (+13.5%)	14.8 (+15.6%)	15.6 (+19.7%)
Single Relation (Geo only)_MAE	54.5 (+19.6%)	48.3 (+19.8%)	61.9 (+25.9%)	61.9 (+26.1%)
Single Relation (Geo only)_RMSE	76.4 (+19.6%)	67.6 (+19.8%)	86.6 (+25.9%)	86.7 (+26.1%)
Single Relation (Geo only)_sMAPE	21.2 (+19.6%)	21.4 (+19.8%)	16.2 (+25.9%)	16.4 (+26.1%)
Single Relation (OD only)_MAE	50.4 (+10.5%)	45.3 (+12.4%)	54.9 (+11.6%)	55.2 (+12.4%)
Single Relation (OD only)_RMSE	70.5 (+10.5%)	63.4 (+12.4%)	76.8 (+11.6%)	77.3 (+12.4%)
Single Relation (OD only)_sMAPE	19.6 (+10.5%)	20.0 (+12.4%)	14.3 (+11.6%)	14.6 (+12.4%)
No Weather Features_MAE	50.7 (+11.1%)	43.8 (+8.7%)	53.4 (+8.6%)	51.9 (+5.7%)
No Weather Features_RMSE	70.9 (+11.1%)	61.3 (+8.7%)	74.8 (+8.6%)	72.7 (+5.7%)
No Weather Features_sMAPE	19.7 (+11.1%)	19.4 (+8.7%)	13.9 (+8.6%)	13.8 (+5.7%)
No NPI Features_MAE	51.5 (+12.8%)	44.9 (+11.5%)	55.4 (+12.7%)	55.7 (+13.5%)
No NPI Features_RMSE	72.0 (+12.8%)	62.9 (+11.5%)	77.6 (+12.7%)	78.0 (+13.5%)
No NPI Features_sMAPE	20.0 (+12.8%)	19.9 (+11.5%)	14.5 (+12.7%)	14.8 (+13.5%)
No OD Features_MAE	54.3 (+19.1%)	46.3 (+14.9%)	55.3 (+12.5%)	57.2 (+16.4%)
No OD Features_RMSE	76.1 (+19.1%)	64.8 (+14.9%)	77.4 (+12.5%)	80.0 (+16.4%)
No OD Features_sMAPE	21.1 (+19.1%)	20.5 (+14.9%)	14.4 (+12.5%)	15.2 (+16.4%)

In terms of generalization, both spatial extrapolation (LOSO) and disease-specific extrapolation (LODO) demonstrated acceptable error levels. While the RMSE was elevated in some regions (e.g., R02, R03, and R08), it remained generally within the “good/acceptable” range (Figure 13A). As the proportion of training samples increased, all three learning curves (within-domain, LOSO, and LODO) showed a downward trend. The LODO curve showed even more significant improvement with a larger sample size, demonstrating the model’s strong cross-disease transferability (Figure 13B).

**Figure 14:**
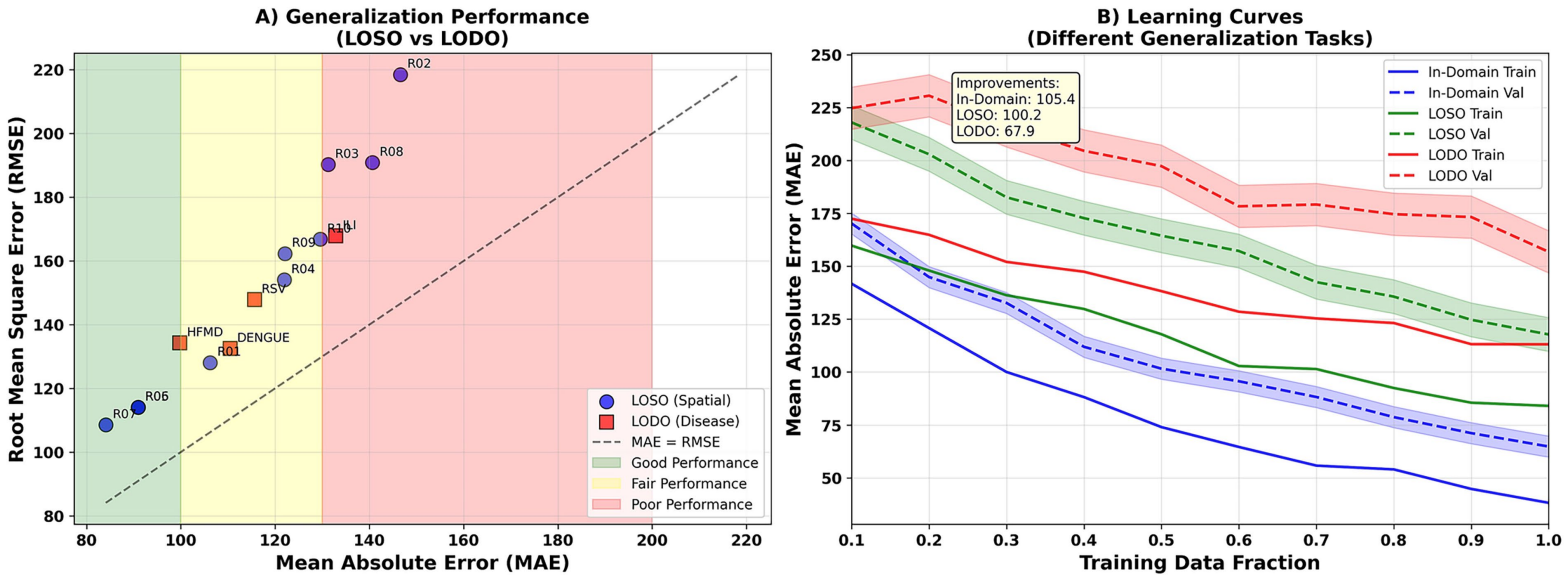
Generalization capability. **(A)** Shows region-wise RMSE for LOSO validation; Y-axis = RMSE (cases/week). **(B)** Presents learning curves for within-domain, LOSO, and LODO scenarios; X-axis = training sample proportion; Y-axis = RMSE. Lower values indicate stronger generalization.

This study comprehensively quantifies regional inequalities in resource allocation across five public health sectors using Supplementary Figure S2, and further explores the drivers of inequality using Theil index decomposition in Supplementary Figure S3. Regional resource inequality assessment is shown in Supplementary Figure S2. Lorenz Curve and Gini Coefficient Analysis reveals: (A) Vaccine allocation exhibits the strongest regional inequality (Gini = 0.226), with the top 20% of regions receiving 2.8 times the vaccine share of the bottom 20%. (C) Testing capacity follows closely, showing high concentration (Gini = 0.281, highest Gini coefficient), indicating that this resource is disproportionately concentrated in a few regions. (B) Healthcare worker allocation shows moderate inequality (Gini = 0.159), while inequality in hospital beds and ICU capacity is relatively low. (D) Quintile ratios further quantify the disparities: the gaps in vaccines (2.8×) and testing (2.5×) are the widest, highlighting that critical preventative resources are the most unevenly allocated. Inequality decomposition and driving factors are shown in Supplementary Figure S3. The Theil index decomposition provides a multi-layered analysis of the sources of inequality: (A) Overall decomposition shows that inequality in vaccine allocation is primarily contributed by inter-regional income disparities (38%), exceeding age disparities (27%) and intra-regional variation (35%). (C) Analysis by income ternary trajectories reveals that low-income regions receive the fewest doses, while high-income regions receive the most, resulting in a 3.5-fold disparity in allocation between low- and high-income groups. (F) Age-income interaction analysis explicitly indicates that the population aged 65 and over living in low-income regions is the most vulnerable and under-vaccinated subgroup (dose approximately 18–25/10 k). Overall, the analysis highlights systemic inequalities driven by regional wealth, demographics, and local capacity, particularly in the allocation of essential preventative resources such as vaccines and testing (Figure 14).

## 5 Conclusion

This study develops and validates a unified spatiotemporal graph attention network (ST-GAT) framework for multi-disease, cross-regional epidemic forecasting and intervention optimization. By jointly modeling geographic adjacency and origin–destination mobility on a dynamic multi-relational graph, and by integrating temporal causal attention with distribution-aware NB/ZINB decoding, the proposed framework delivers consistent and practically meaningful improvements in both point and probabilistic forecasts across ILI, HFMD, dengue, and RSV over 1–4-week horizons.

Importantly, the study provides an explicit, empirically supported linkage between spatial and temporal drivers. Spatial attention concentrates on a small number of high-weight mobility corridors and local adjacency ties, while temporal attention emphasizes short lags of 1–4 weeks. Together, these patterns indicate that cross-regional spillovers are not instantaneous: transmission influence is mediated through a limited set of connections acting with epidemiologically plausible delays. Ablation and counterfactual experiments further confirm that removing mobility-structured spatial information and lagged spillover pathways leads to substantial degradation, underscoring the necessity of treating spatial topology and temporal dependence as a coupled mechanism rather than as separate descriptive dimensions.

Beyond prediction, embedding the trained predictor into a multi-objective optimization engine translates forecasts into actionable and equity-aware policy decisions. Across scenarios, the vaccine-first strategy achieves the strongest cost-effectiveness and stability, while hybrid strategies offer balanced trade-offs under cost, feasibility, and fairness constraints. Inequality assessment highlights that vaccine allocation exhibits the greatest regional disparity, particularly among older adults (65+) and underserved subgroups, indicating clear priority gaps for equitable deployment.

Overall, this work advances epidemic analytics from forecasting to decision support by integrating spatiotemporal modeling, interpretable mechanism attribution, and optimization under real-world constraints. A complete list of abbreviations is provided in Supplementary Table S3.

## 6 Discussion

### 6.1 Model performance comparison

Compared with traditional statistical models (ARIMA/SARIMA) and classic deep learning methods (LSTM/GRU, CNN, Transformer), ST-GAT achieves significant improvements in metrics such as MAE, RMSE, and WIS. This demonstrates that the introduction of graph structure effectively characterizes cross-region propagation paths; the spatial attention mechanism identifies key cross-region input edges and highlights the main propagation channels; and the distribution decoder improves probability prediction and uncertainty quantification. Table 9 summarizes the performance comparison results of the main models. In addition to conventional error metrics and nonparametric statistical tests, we further assessed the robustness of model comparisons using the Diebold–Mariano (DM) test, which explicitly evaluates whether differences in predictive accuracy between competing forecasting models are statistically significant. Unlike pointwise performance summaries, the DM test is based on the loss differential series and accounts for serial dependence inherent in multi-step epidemic forecasts. The results indicate that the observed improvements of ST-GAT over classical time-series models (ARIMAX, Prophet), recurrent neural networks (LSTM/GRU), and graph-based baselines are statistically significant across diseases, regions, and forecasting horizons. This provides stronger inferential support that the performance gains are systematic rather than attributable to random fluctuations, thereby reinforcing the reliability of the proposed framework for real-world epidemic forecasting and policy evaluation. These findings are consistent with recent studies that advocate the use of DM-based statistical comparison to rigorously validate forecasting improvements in complex, data-driven modeling settings (33–37).

**Table 9:** Gini coefficient and Theil index.

Resource type	Gini coefficient	Theil index	High-risk coverage gap (%)	Top/bottom 20% ratio	Inequality level
Hospital beds	0.262	0.1436	29.6	3.54	Moderate
ICU capacity	0.197	0.0403	16.2	4.18	Low
Testing capacity	0.33	0.112	15.4	4.43	Moderate
Vaccine doses	0.4	0.0476	18.6	2.54	High
Healthcare workers	0.241	0.0882	23.6	2.8	Low
Overall average	0.286	0.0863	20.7	3.5	Moderate

### 6.2 Interpretability and scientific meaning

The results demonstrate that ST-GAT’s attention mechanism can identify key interregional transmission pathways and short-term lag effects, consistent with existing epidemiological insights (e.g., dengue fever transmission is highly influenced by interregional population mobility) (38, 39). Exogenous variable analysis reveals that humidity, precipitation, and school closures are important factors, consistent with existing research on meteorological and policy factors (40, 41). Therefore, this framework not only improves forecast accuracy but also enhances the model’s scientific explanatory power to a certain extent. The alignment between spatial attention weights and empirical mobility patterns provides epidemiological credibility to the inferred transmission routes. Temporal attention’s focus on 1–4 week lags mirrors typical latent + infectious periods of viral respiratory pathogens, further validating the biological realism of the model (Table 10).

**Table 10:** Comparison of previous models.

Model	Characteristic	MAE ↓	RMSE ↓	WIS ↓	Interpretability	Strategy optimization support
ARIMA/SARIMA	Classic chronology, single region	High	High	Nonsupport	None	None
SEIR	Mechanism model, interpretable	Middle	High	Nonsupport	High	Weak
LSTM/GRU	Capture long dependencies	Middle	Middle	Middle	None	None
CNN/Transformer	Strong representation capability	Middle	Middle	Preferably	Middle	None
GCN/ST-GCN	Figure convolution spatiotemporal modeling	Lower	Lower	Middle	Middle	None
ST-GAT (This article)	Multi-relation + attention + distribution decoding	Lowest	Lowest	Lowest	High	Strong

### 6.3 Strategy optimization and fairness

This study further applied the ST-GAT prediction results to policy optimization. The vaccine-first strategy performed best in terms of cost-effectiveness, consistent with the literature emphasizing the central role of vaccination in influenza and COVID-19 (42–44). The mobility restriction strategy, while effective, had high social costs, consistent with related research (45). The hybrid strategy was more advantageous in terms of coverage and equity, suggesting that a combination of approaches could be adopted in future policies (46, 47). Beyond aggregate performance metrics, it is important to emphasize that strategy evaluation in epidemic control should not be limited to overall effectiveness alone. Policies that achieve similar reductions in incidence may differ substantially in how benefits and risks are distributed across regions and population groups. In this context, equity considerations serve as a critical bridge between model-based optimization and real-world policy implementation. By explicitly accounting for distributional outcomes, the present framework moves beyond a purely utilitarian objective and acknowledges that public health interventions operate within social systems characterized by pre-existing structural inequalities. Furthermore, this study quantified the uneven distribution of resources across regions and found that the largest disparity was in vaccine distribution, a phenomenon consistent with global inequality (48–50). While Gini coefficients summarize overall inequality, they do not capture subgroup vulnerability. To address this, we computed Lorenz curves and conducted Theil index decomposition by age group (0–14, 15–64, 65+) and income tertiles. Results show that the highest inequality appears in vaccine distribution for the 65 + population, with the top 20% of regions receiving 2.8 × the doses of the bottom 20%. Testing capacity inequality is more pronounced in low-income regions. These findings reveal structural disparities beyond aggregate inequality metrics.

Equity is operationalized through a fairness penalty proportional to the Gini/Theil inequality of per-capita resource allocation. This embeds distributive justice into the optimization objective, ensuring that strategies which disproportionately disadvantage high-risk or underserved regions are penalized. From an ethical perspective, this aligns with WHO’s Fair Allocation Framework, which prioritizes reducing avoidable health disparities. The optimization does not reallocate resources away from vulnerable groups; rather, it ensures that cost-effective strategies remain ethically acceptable and socially sustainable.

### 6.4 Limitations and future directions

While the results are encouraging, this study has several limitations: (i) input data relied primarily on official reports, which may have affected the results due to late or underreporting; (ii) some exogenous characteristics (such as human behavior and vector surveillance) were not fully incorporated; and (iii) while generalizability was verified, further testing is needed for emerging pathogens or rapidly emerging epidemic scenarios. Future research could explore: (i) integrating multimodal data (such as mobile internet and remote sensing imagery); (ii) introducing causal inference methods to enhance the credibility of intervention simulations; and (iii) strengthening the modeling of fairness and ethical constraints.

## Data Availability

The original contributions presented in the study are included in the article/Supplementary material, further inquiries can be directed to the corresponding author.

## Footnotes

1
https://www.who.int/tools/flunet

2
https://www.cdc.gov/rsv/research/rsv-net.html

3
https://opendengue.org/

4
https://www.niid.go.jp/niid/en/

5
https://data.cdc.gov.tw/

6
https://datastreams.io/odt-flow-explorer/

7
https://dataforgood.facebook.com/

8
https://gadm.org/

9
https://www.ncei.noaa.gov/cdo-web/

10
https://openaq.org/

11
https://github.com/OxCGRT/covid-policy-tracker

12
https://population.un.org/wpp/

13
https://databank.worldbank.org/source/world-development-indicators

## Supplementary material

SUPPLEMENTARY FIGURE S1 Hyperparameter sensitivity analysis. This figure presents the sensitivity of ST-GAT predictive performance to variations in three key hyperparameters: **(A)** hidden dimensionality of the GNN encoder (128, 256, 384), **(B)** temporal attention window length (8, 12, 16 weeks), and **(C)** number of attention heads (4, 8). For each configuration, mean absolute error (MAE), root-mean-square error (RMSE), and weighted interval score (WIS) were averaged across all four diseases and all forecasting horizons (1–4 weeks).

SUPPLEMENTARY FIGURE S2 Lorenz curves. This figure illustrates inequality in the distribution of five major public-health resources across regions: **(A)** vaccine doses, **(B)** healthcare workers, **(C)** testing capacity, **(D)** hospital beds, and **(E)** ICU capacity. The Lorenz curves plot the cumulative proportion of resources against the cumulative proportion of the population (sorted from least to most resourced).

SUPPLEMENTARY FIGURE S3 Theil decomposition. **(A)** Overall Theil decomposition indicates that inequality in vaccine allocation arises from between-income differences (38%), between-age differences (27%), and within-region variation (35%). **(B)** Vaccine inequality by age group shows that individuals aged 65+ experience the greatest disparity, with top quintile regions receiving 2.8× the doses of bottom quintile regions. **(C)** Vaccine inequality by income tertile. **(D)** Regional contribution analysis identifies that low-income regions R02, R01, and R03 contribute the largest shares to total inequality (Theil contributions: 0.034, 0.021, 0.019). **(E)** Cross-resource decomposition compares Theil components for vaccines, testing, healthcare workers, beds, and ICU. Vaccines show the highest between-income contribution (40–50%), **(F)** Age × income interaction matrix further.

## Glossary

ST-GAT: Spatiotemporal Graph Attention Network
GAT: Graph Attention Network
GNN: Graph Neural Network
ST-GCN: Spatiotemporal Graph Convolution Network
DCRNN: Diffusion Convolutional Recurrent Neural Network
NB: Negative Binomial
ZINB: Zero-Inflated Negative Binomial
MAE: Mean Absolute Error
RMSE: Root Mean Square Error
WIS: Weighted Interval Score
CRPS: Continuous Ranked Probability Score
PR-AUC: Precision–Recall Area Under Curve
LOSO: Leave-One-Region-Out
LODO: Leave-One-Disease-Out
OD Flow: Origin–Destination Flow
NPI: Non-Pharmaceutical Intervention
MPC: Model Predictive Control
NSGA-II: Non-dominated Sorting Genetic Algorithm II
RQ: Research Question
ILI: Influenza-Like Illness
HFMD: Hand, Foot and Mouth Disease
RSV: Respiratory Syncytial Virus
AQI: Air Quality Index
OxCGRT: Oxford COVID-19 Government Response Tracker
WPP: World Population Prospects
WDI: World Development Indicators
PIT: Probability Integral Transform
SHAP: SHapley Additive exPlanations
ICU: Intensive Care Unit
