# Supplementary figures and images for: Dynamic spatiotemporal graph attention networks for cross-regional multi-disease forecasting and intervention optimization
Source: Front Public Health. 2026 Feb 4;14:1720620. doi: 10.3389/fpubh.2026.1720620 (PMC12913526; doi:10.3389/fpubh.2026.1720620)

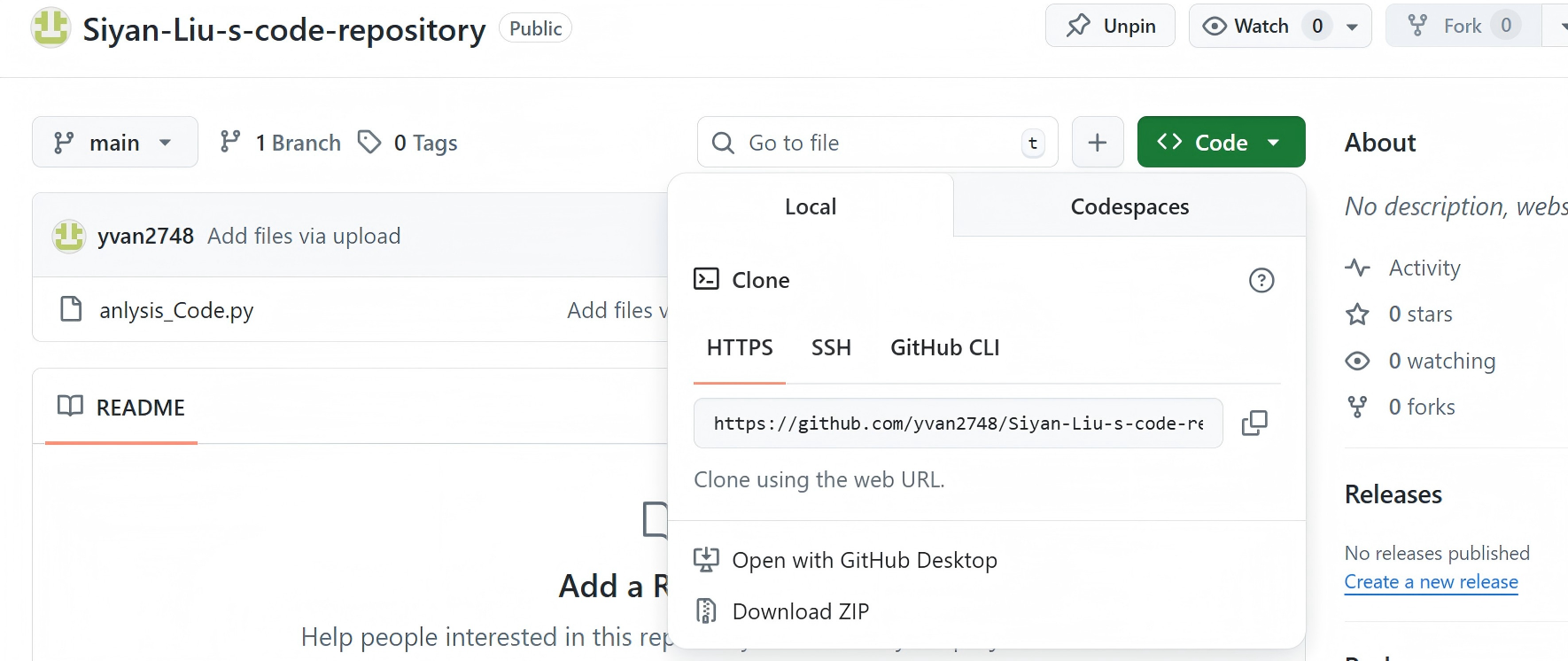

Supplement: SUPPLEMENTARY FIGURE S1 — Hyperparameter sensitivity analysis. This figure presents the sensitivity of ST-GAT predictive performance to variations in three key hyperparameters: (A) hidden dimensionality of the GNN encoder (128, 256, 384), (B) temporal attention window length (8, 12, 16 weeks), and (C) number of attention heads (4, 8). For each configuration, mean absolute error (MAE), root-mean-square error (RMSE), and weighted interval score (WIS) were averaged across all four diseases and all forecasting horizons (1–4 weeks). [file Image_1.PNG]

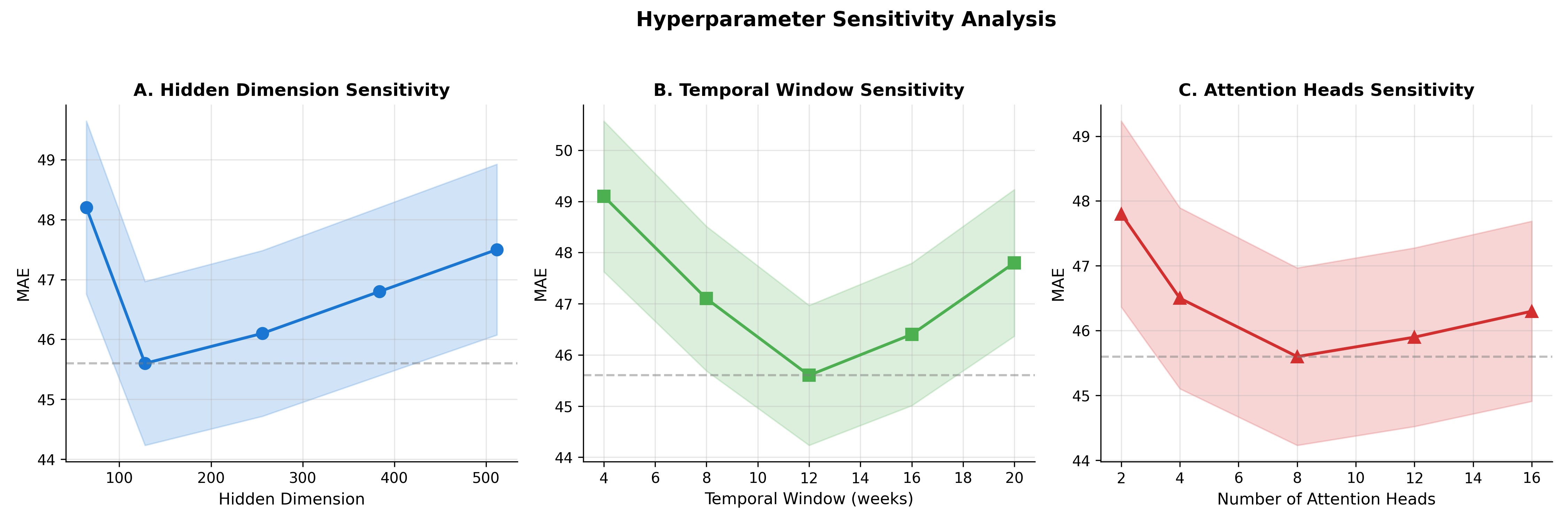

Supplement: SUPPLEMENTARY FIGURE S2 — Lorenz curves. This figure illustrates inequality in the distribution of five major public-health resources across regions: (A) vaccine doses, (B) healthcare workers, (C) testing capacity, (D) hospital beds, and (E) ICU capacity. The Lorenz curves plot the cumulative proportion of resources against the cumulative proportion of the population (sorted from least to most resourced). [file Image_2.JPEG]

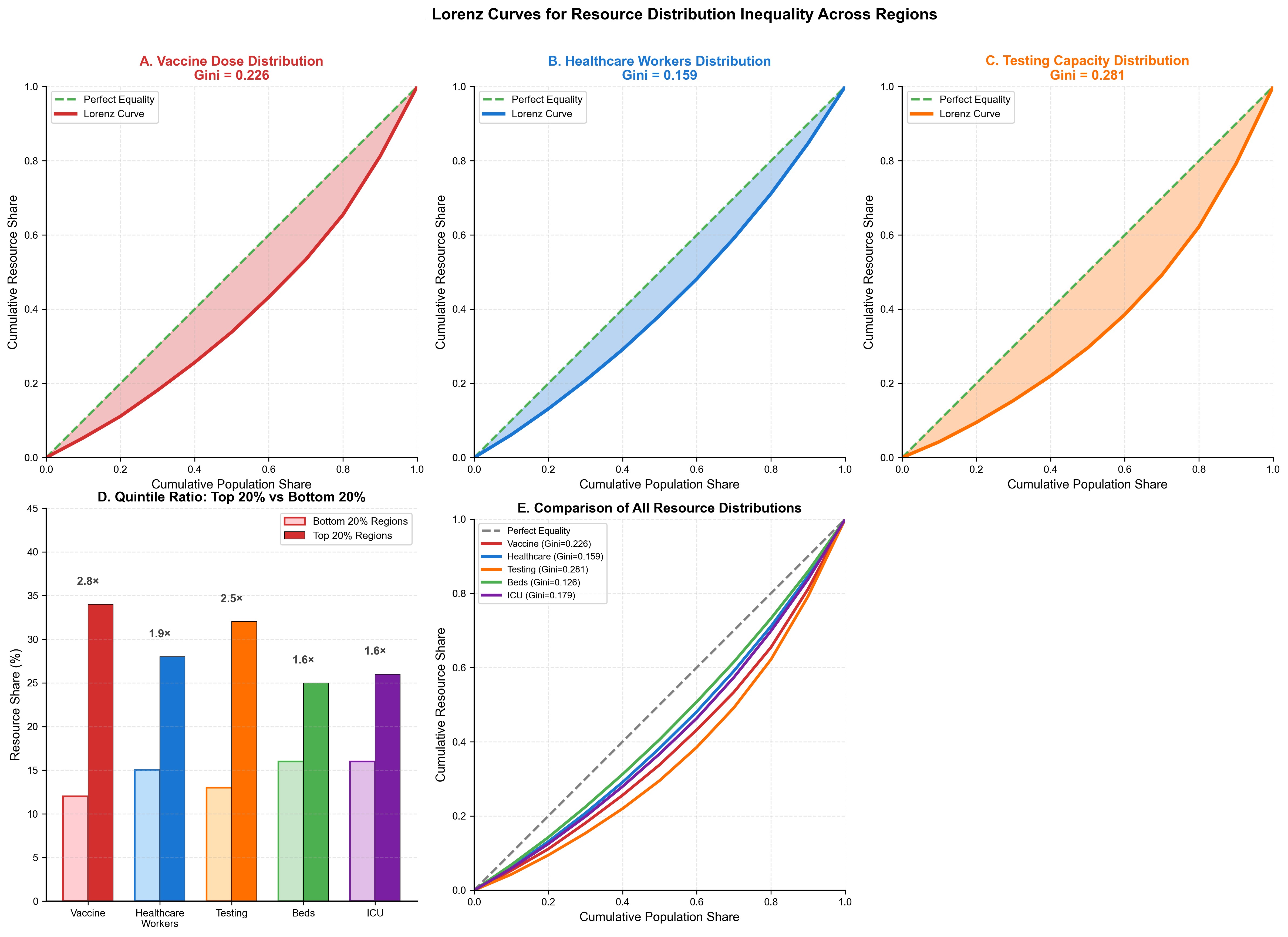

Supplement: SUPPLEMENTARY FIGURE S3 — Theil decomposition. (A) Overall Theil decomposition indicates that inequality in vaccine allocation arises from between-income differences (38%), between-age differences (27%), and within-region variation (35%). (B) Vaccine inequality by age group shows that individuals aged 65+ experience the greatest disparity, with top quintile regions receiving 2.8× the doses of bottom quintile regions. (C) Vaccine inequality by income tertile. (D) Regional contribution analysis identifies that low-income regions R02, R01, and R03 contribute the largest shares to total inequality (Theil contributions: 0.034, 0.021, 0.019). (E) Cross-resource decomposition compares Theil components for vaccines, testing, healthcare workers, beds, and ICU. Vaccines show the highest between-income contribution (40–50%), (F) Age × income interaction matrix further. [file Image_3.JPEG]

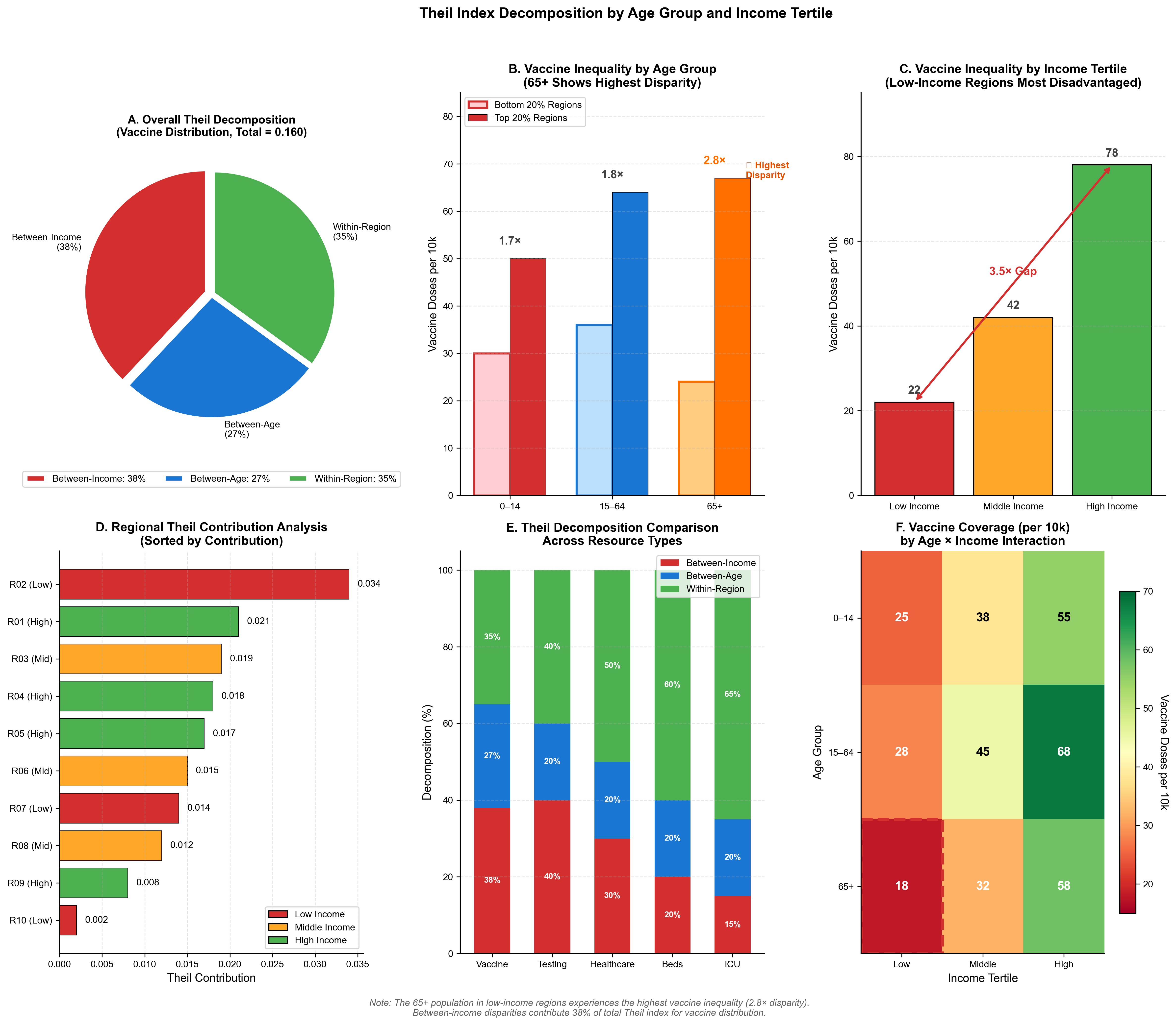

Supplement: Supplementary file 4 [file Image_4.JPEG]
